# DeepSAT: Learning Molecular Structures from Nuclear Magnetic Resonance Data

**DOI:** 10.1186/s13321-023-00738-4

**Published:** 2023-08-07

**Authors:** Hyun Woo Kim, Chen Zhang, Raphael Reher, Mingxun Wang, Kelsey L. Alexander, Louis-Félix Nothias, Yoo Kyong Han, Hyeji Shin, Ki Yong Lee, Kyu Hyeong Lee, Myeong Ji Kim, Pieter C. Dorrestein, William H. Gerwick, Garrison W. Cottrell

**Affiliations:** 1 Center for Marine Biotechnology and Biomedicine, Scripps Institution of Oceanography, University of California San Diego, La Jolla, CA USA; 2 College of Pharmacy and Integrated Research Institute for Drug Development, Dongguk University-Seoul, Gyeonggi-Do, Republic of Korea; 3 Department of Computer Science and Engineering, University of California, La Jolla, San Diego, CA USA; 4 Institute of Pharmaceutical Biology and Biotechnology, University of Marburg, Marburg, Germany; 5 Skaggs School of Pharmacy and Pharmaceutical Sciences, University of California San Diego, La Jolla, CA USA; 6 Ometa Labs LLC, San Diego, CA USA; 7 Department of Computer Science, University of California Riverside, Riverside, CA USA; 8 Department of Chemistry and Biochemistry, University of California San Diego, La Jolla, CA USA; 9 Institut de Chimie de Nice, UMR 7272, Université Côte d’Azur, CNRS, 06108 Nice, France; 10 College of Pharmacy, Korea University, Sejong, Republic of Korea

**Keywords:** Convolutional neural network, Nuclear magnetic resonance, Structure prediction

## Abstract

The identification of molecular structure is essential for understanding chemical diversity and for developing drug leads from small molecules. Nevertheless, the structure elucidation of small molecules by Nuclear Magnetic Resonance (NMR) experiments is often a long and non-trivial process that relies on years of training. To achieve this process efficiently, several spectral databases have been established to retrieve reference NMR spectra. However, the number of reference NMR spectra available is limited and has mostly facilitated annotation of commercially available derivatives. Here, we introduce DeepSAT, a neural network-based structure annotation and scaffold prediction system that directly extracts the chemical features associated with molecular structures from their NMR spectra. Using only the ^1^H-^13^C HSQC spectrum, DeepSAT identifies related known compounds and thus efficiently assists in the identification of molecular structures. DeepSAT is expected to accelerate chemical and biomedical research by accelerating the identification of molecular structures.

**Supplementary Information:**

The online version contains supplementary material available at 10.1186/s13321-023-00738-4.

## Introduction

Small molecules are generally defined as any organic compound with low molecular weight (≤ 900 Da). Small molecules have been an important source of lead compounds for drug discovery and medicinal applications as a result of their structural diversity and potent biological activities [1]. Nowadays, small molecules contribute more than half of pharmaceutical drugs currently marketed; this has occurred for diverse medical conditions including cancer, microbial infections, viral diseases, hyperlipidemia, diabetes, and many others [2, 3]. The chemical diversity of small molecules, also known as ‘chemical space’, continues to expand as a result of contributions from organic synthesis or natural products (NP) discovery. For instance, there are over 326,000 NPs reported from terrestrial and marine organisms, and on average there are 1600 new marine and microbial NPs reported annually [4, 5].

Identification of molecular structure is an essential aspect of small molecule-based drug discovery. However, this intensive and time-consuming activity can be costly and strongly dependent upon a researcher’s expertise. To avoid rediscovering known compounds, a variety of methodologies have been developed at different stages of the isolation and identification process [6]. For example, the Global Natural Products Social (GNPS) Molecular Networking tool enables the matching of fragmentation spectra from mass spectrometry experiments (MS) allowing researchers to annotate the metabolites in mixtures [7]. Other MS-based annotation approaches have employed statistical or machine learning-based approaches to locate and target the isolation of novel chemical entities [8–11]. Alternatively, genome mining approaches employ bioinformatic tools such as AntiSMASH [12] and BiG-SCAPE/CORASON [13] to gain insight into the chemical nature of NPs from genetic sequence information. These strategies accelerate the annotation of known compounds and can inform the targeted discovery of novel ones. Nevertheless, the precise structure elucidation of an unknown compound still requires isolation and NMR experiments which can represent a great deal of time, depending on the investigator’s experience [14].

The complete structure elucidation of novel molecules typically requires several types of information, including NMR, MS, UV, IR, and ECD, as well as modifications from chemical reactions [15]. Among the spectroscopic methods, NMR experiments are central to establishing molecular structure, as they can reveal atom relationships through bonds as well as through space [16, 17].

Known compounds can be identified by comparing their 1D ^1^H and ^13^C NMR data to reference spectra in the literature along with their molecular weight information. However, those reference spectra are highly dispersed such that searching and comparing with reference spectra is a challenging process. To improve the retrieval of reference NMR spectra, open-sourced spectral libraries have been introduced, such as NMRShiftDB (n = 53,954 reference spectra) [18], BioMagResBank (n = 11,900) [19], HMDB (n = 4036) [20], CH-NMR-NP (n = 35,500) [21], the NP-MRD (n = 19,840) [22], and CSEARCH (n = 340,554) [23]. These databases provide ^1^H, ^13^C and/or 2D NMR data for NPs and other metabolites. Additionally, comprehensive spectral reference search tools such as MetaboMiner (n = 502) and COLMAR (n = 701) were developed in order to search 1D and 2D NMR data with reference spectra from important metabolites [24, 25]. Nonetheless, the number of chemical entities and the structure diversity in these databases is limited and does not cover the enormous chemical diversity of nature.

To overcome this lack of reference data, commercial and non-commercial computer assisted structure elucidation (CASE) tools have been developed, such as the ACD/structure elucidator (ACD/Labs), CMC-se (Bruker), the MNOVA structure elucidation tool (Mestrelab), and LSD [26, 27]. Using CASE programs, the most probable structures are generated by analysis of 1D and 2D NMR data along with molecular formula information for the target molecule. However, confidently identifying the molecular formula of a new molecule often requires high resolution mass spectra, and sometimes such information can be ambiguous or difficult to obtain. Additionally, ^13^C NMR chemical shifts with their associated carbon type (C, CH, CH_2_ and CH_3_), and 2D NMR experiments such as ^1^H-^1^H COSY, ^1^H-^13^C HSQC, ^1^H-^13^C HMBC, and ^1^H-^1^H NOESY are required to establish atom connectivity and propose a structure with high confidence [28]. Kuhn et al. [29, 30] presented the proof-of-concept methods of substructure prediction and compound classification from NMR spectra using a convolutional neural network.

Previously, we introduced SMART 2.0, an artificial intelligence-based tool for retrieving structure candidates from an in-house NMR database called the Moliverse, specifically constructed from ^1^H-^13^C HSQC spectra [31]. SMART 2.0 increased the accuracy of the method compared to the first SMART 1.0 prototype, which was trained using a very limited dataset [32]. Since its introduction, the SMART tools have supported natural products researchers in their discoveries of molecules from marine and terrestrial organisms [33–35]. However, even though SMART 2.0 has shown very good performance over other spectral library retrieval systems, all available NMR spectra in the Moliverse covered around 130,000 compounds. Further expansion of the library is limited because of the unavailability of reference compounds or the extensive time required to accurately calculate large numbers of NMR spectra using quantum mechanics. On the other hand, molecular structure databases such as Pubchem [36] contain millions of compounds. If molecular structures could be searched directly in these databases using NMR-based structural representations as the input, then the coverage of the resulting system would be vastly improved. 

**Fig. 1:**
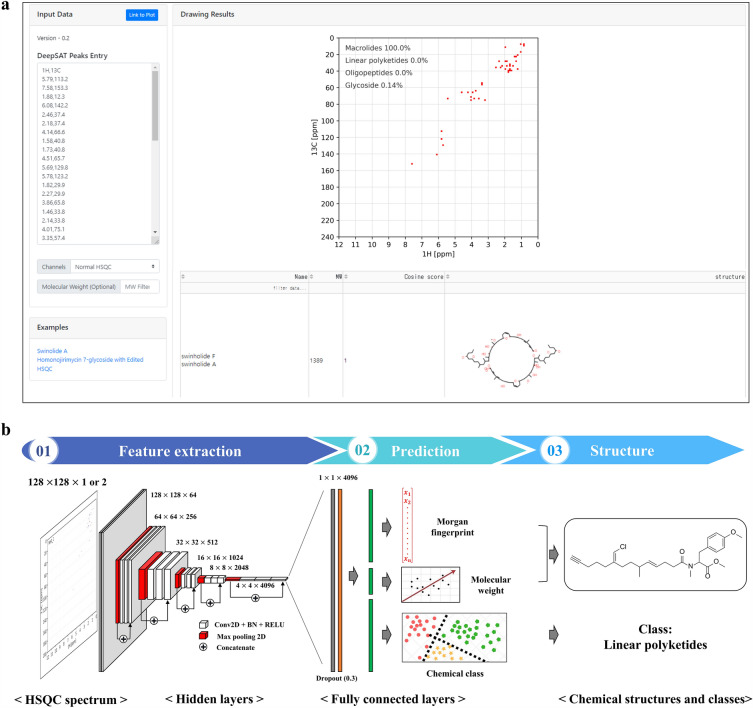
Overview of DeepSAT. **a** Web-based platform of DeepSAT analysis (https://deepsat.ucsd.edu). **b** The multi-task learning architecture of DeepSAT. In the feature extraction step, the convolutional neural network extracts the features from HSQC spectra. Based on the extracted features, fully connected layers predict Morgan fingerprints, molecular weights, and chemical classes. By using the predicted properties, structure annotation is accelerated

Consequently, in this study we introduce DeepSAT (https://deepsat.ucsd.edu), an NMR-based structure searching tool that uses NMR spectra as the user input. In DeepSAT, large numbers of molecules are searchable even if no authentic NMR spectra are available. DeepSAT outperforms all other available NMR-based tools for identification of small molecular structures or for finding similar structures. DeepSAT was trained using a convolutional neural network (CNN)-based multi-task supervised learning architecture with 143,467 ^1^H-^13^C HSQC spectra collected or calculated from diverse molecules. This neural network uses the ^1^H-^13^C HSQC spectra as input and predic its chemical fingerprints, molecular weights, and structure classes of molecules. These three features are then used to search for small molecules with similar chemical characteristics from chemical databases. Thus, DeepSAT has the potential to further accelerate the efficiency and accuracy of structure identification in small molecule-based drug discovery (Fig. 1).

## Materials and method

### NMR data preparation for DeepSAT dataset

The NMR spectra for the training dataset were established from a combination of literature and computed NMR spectra. The literature data was from the CH-NMR-NP database where the ^1^H and ^13^C NMR spectra of 29,500 natural products and 6,000 organic compounds were compiled from published papers. Incomplete or incorrect data were manually filtered. In order to increase the number of spectra for training, computed NMR spectra were generated using ACD/Spectrus Processor 2017.2.1 software (File Version S70S41, Build 99684, 21 Feb 2018; Advanced Chemistry Development, Inc.), in which 113,967 compounds were randomly chosen from the Universal Natural Product Database, NPATLAS (https://www.npatlas.org), NPASS (http://bidd.group/NPASS), GNPS (http://gnps.ucsd.edu), and NPClassifier (http://npclassifier.ucsd.edu). All selected structures were submitted as SMILES strings and their HSQC spectra were calculated by using the corrected weighted average experimental algorithm in the ACD software. The computational parameters were set as follows; ‘correlation’ was set as C-H COSY, ‘experimental’ was set as HSQC-DEPT, ‘spectrometer frequency’ was set to 600 MHz, ‘spectrum size’ was 128 by 128 pixels, ‘spectrum bounds’ was signal-dependent, ‘line width’ was 3 Hz, and ‘solvent’ was chloroform-*d* as default.

### Chemical properties calculation

The Morgan fingerprint method was chosen for the DeepSAT analysis and modified as described below to generate chemical fingerprints using RDKit version 2020.03.2. The range of radius was set from 0 to 2 with hydrogen atoms added to the molecular graphs, and a total of 6144 chemical features were identified for the training. Molecular weights were also calculated using RDKit and rounded up to the second digit after the decimal point. All molecules in this study were classified to “superclass” using the NPClassifier ontology (http://npclassifier.ucsd.edu).

### Convolutional Neural Network Architecture and Hyperparameters

The training of DeepSAT was performed on a server with an Intel^®^ Core™ i7-6850 K CPU, three NVIDIA^®^ GeForce GTX 1080 with 8 GB video memory GPUs, and 64 GB RAM. Python programming was used in this project, and the TensorFlow 2.3.0 deep learning framework was used. The CNN for DeepSAT was comprised of two different networks that were designed for normal HSQC and multiplicity-edited HSQC, respectively. The convolutional layers along with the fully connected ones were the same in both networks with different input shapes; the normal HSQC had a shape of (128,128,1) whereas the Edited HSQC had a shape of (128,128,2). A dropout layer was applied to the global max pooling layer to improve generalization. The activation function for the hidden layers used the ReLU function and all hidden layers were normalized by batch normalization to avoid overfitting and vanishing gradients. Hyperparameters for training the deep neural networks for DeepSAT were set as follows: the optimizer was Adam with a learning rate of 10^–5^ (decay = 10^–6^). Activation functions were ReLU (hidden layers), sigmoid (fingerprint prediction layer), and softmax (classification layer). Loss functions were binary cross entropy (fingerprint prediction layer), sparse categorical cross entropy (classification layer), and mean absolute percentage error (molecular weight prediction layer). Dropout rate was 0.2 and Batch size was 16 (See Additional file 1).

### Evaluation

For evaluation, 3982 HSQC spectra were randomly chosen for the test set and separated from the training and validation dataset. The test set was used to evaluate the prediction performance of DeepSAT in comparison with the performance of other available tools, including SMART 2.0 and NMRShiftDB. For searching NMRShiftDB with queried NMR data, a Python script was established to automate the search process and all queried data were established from the HSQC data. For searching ^1^H NMR spectra from NMRShiftDB, the search type was set as ^1^H in the complete mode. For the search of ^13^C NMR spectra, the search type was set as ^13^C in the subspectrum mode because HSQC spectra provide only partial ^13^C data. All results from NMRShiftDB were sorted by similarity scores calculated by the database. The structure similarities were calculated using the chemical fingerprint method with cosine scoring. The threshold values were set as 1.0 for identical compounds and 0.8 for the similar compounds, and these values were used to evaluate identification and annotation rates, respectively. The correct identification and annotation rates at top *k* were computed by percentage of correctly identified or annotated structures found in the top *k* output. The precision@*k*, recall@*k*, and F1-score@*k* of structure annotation were calculated from the annotation results. The definition of precision@*k* in this study is:

$$Precision@k = \frac{\# Of \,correctly \,annoated \,structures\, at \,k}{k}$$

The recall@*k* is defined as the percentage of correctly annotated structures from all similar structures in the database when *k* structures were annotated. The exact definition of recall@*k* is:

$$Recall@k = \frac{\# Of \,correctly \,annotated \,structures\, at\, k}{\# Of \,similar \,structures \,in \,the \,database}$$

F1-score@*k* defined as the harmonic mean of precision and recall.

**Evaluation of Different Solvents on DeepSAT Predictions**

In order to evaluate for the experiment evaluating DeepSAT’s sensitivity to the solvent used, we obtained 36 HSQC spectra for the same 18 NPs dissolved in two solvents: methanol-d4 (n = 18) and chloroform-d (n = 18). NMR spectra were measured using a Bruker SPECTROSPIN 600 spectrometer equipped with 5 mm probes. Compounds were dissolved in 0.6 mL of chloroform-d or methanol-d4. HSQC spectra were measured at room temperature (298.15 oK, 25.0 °C). NMR experiments were performed using standard Bruker pulse programs (XWinNMR). HSQC spectra were obtained using the Bruker library pulse sequence ‘hsqcetgpsi’ conditions: ns 16, d1 1.5 s, SWH 12019.23 Hz and td = 1024.

## Results

**Chemical properties can be accurately predicted from NMR spectra.**

**Fig. 2:**
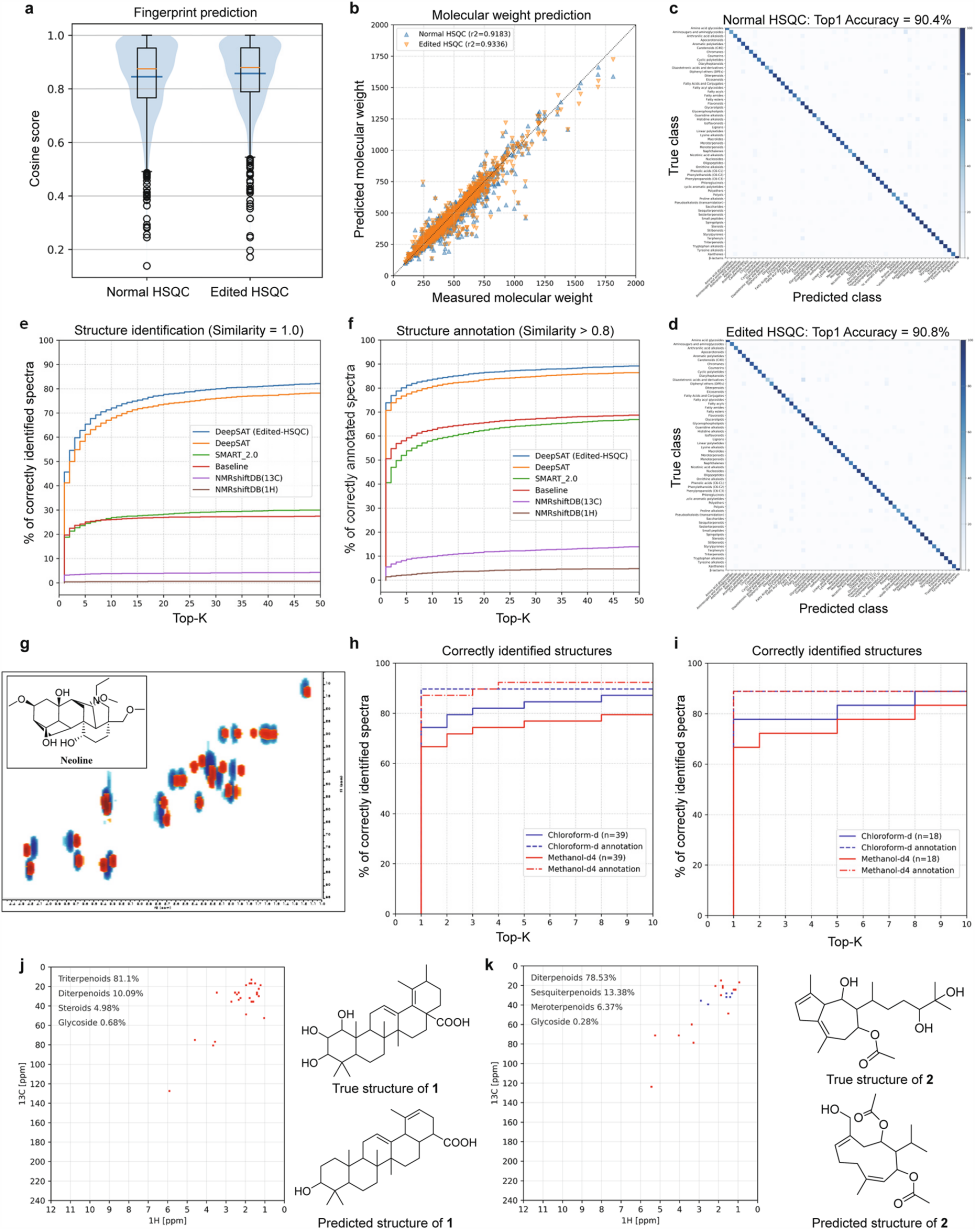
Evaluation of DeepSAT using a test set. **a** Average (orange line) and median (blue line) of cosine scores between predicted and ground truth fingerprints for HSQC and Edited HSQC data input. **b** Linear regression between measured (x axis) and predicted molecular weights (y axis). **c** and **d** Confusion matrix of classification results using DeepSAT with normal HSQC data and multiplicity edited HSQC data. **e** Percentage of correctly identified structures in the top k output of several different tools, for maximum rank k = 1, 2, …, 50. **f** Percent of correctly annotated structures in the top k. For the measurement of annotation rate, cosine score of 0.8 was set as the threshold. **g** Experimental HSQC spectrum of the natural product, neoline dissolved in chloroform-d (blue) and methanol-d_4_ (red). **h** Identification (solid) and annotation (dashed) rates in total experimental data. **i** Identification (solid) and annotation (dashed) rates in compounds with NMR data recorded in both solvents. **j** and **k** HSQC spectra and predicted results of previously undescribed compounds **1** and **2**

To evaluate the performance of DeepSAT, we established a test set (n = 3982) of HSQC spectra that were randomly chosen and excluded from the training of SMART 2.0 and other tools used in the evaluation. Evaluation of the performance of DeepSAT predictions was carried out in two ways. First, the chemical fingerprint prediction, molecular weight prediction, and structure classification were evaluated by specific metrics. Second, the identification and annotation results were benchmarked with the other methods (Fig. 2).

As the fingerprints predicted from our method consisted of strings containing 6144 binary bits, cosine similarity was used as the metric for measuring the similarity between predicted fingerprints and actual ones. As shown in Fig. 2a, the average cosine score was 0.8450 for the normal HSQC model, and 0.8574 for the Multiplicity Edited HSQC model. The molecular weight prediction results are represented by R-squared values, and for the normal and multiplicity edited HSQC models the results were 0.9183 and 0.9336, respectively (Fig. 2b). Finally, we evaluated and analyzed the classification performance using an accuracy metric and a confusion matrix for the 59 structure class categories from the NPClassifier ontology. In the compound class prediction from HSQC spectra, the Top-1 accuracy of normal and Edited HSQC models were 90.4% and 90.8%, respectively. To further evaluate the performance of the network, we created confusion matrices where the rows correspond to the ground-truth classes and the columns correspond to the predicted class. We show these in Fig. 2c, d (enlarged versions of these are in the supplementary material). As can be seen from the figure, the diagonal elements generally showed high values, indicating excellent performance. The F1-scores of the classifier on each element are given in the supplementary material in Tables S2 and S3. Several compound classes, such as diazotetronic acids and derivatives (F1-score of 0.64) and naphthalenes (F1-score of 0.66), showed low values. These results appear to be related to their proton-deficient nature (diazotetronic acids and derivatives) or that the structural category is too broad (naphthalenes). The precision and recall of classification and glycoside prediction are provided in Table 1. As expected, the multiplicity edited HSQC-based model showed slightly better results than the normal HSQC model in predicting compound class (Supplemental File S17). However, predictions of the presence of a glycoside were similar in both experiments.

 In the molecular structure search, our new method using predicted chemical properties including chemical fingerprints, molecular weights and compound classes outperformed all other existing tools of a similar nature (Fig. 2e). Compared with SMART 2.0, the number of correct identifications was over 2-fold higher (41.0% vs 18.7%) when using normal HSQC spectra (SMART 2.0 does not support multiplicity edited HSQC data). In the Top-5 outputs, DeepSAT with standard HSQC spectra achieved 60.6% correct identifications. When using multiplicity edited HSQC as the input, this was increased to 45.2% as the Top-1 output and 64.9% in the top 5 outputs. The results from searching NMRshiftDB by proton and carbon NMR shifts, however, achieved under 3% of correct identifications. As a baseline against which to compare the performance of DeepSAT, the in-house reference HSQC spectral library (n=143,467) was used to retrieve candidate molecular structures by a simple matching of the query compound chemical shifts with those in the library. The thresholds for the peak shift differences were set as 0.5 ppm for carbon signals and 0.05 ppm for proton signals. This baseline analysis gave nearly equal results to SMART 2.0, and overall showed that the reengineered implementation of DeepSAT greatly improved the correct identification rate of the predicted structures and properties.

**Table 1:** The precision and recall rates of classification and glycosides prediction results (n = 3982)

Model	Classification			Glycosides		
	Precision	Recall	F1-score	Precision	Recall	F1-score
DeepSAT (Normal HSQC)	0.8788	0.8733	0.8726	**0.9372**	**0.9376**	**0.9364**
DeepSAT (Multiplicity Edited HSQC)	**0.9120**	**0.9047**	**0.9046**	0.9315	0.9284	0.9251

Best values in each column are bolded

While identifying known molecules is of paramount importance, structure annotation of novel structures is also important, as this can facilitate structure elucidation by allowing comparison of spectroscopic data sets with previously described molecules. Generally, we consider two structures to be “similar” if the cosine score is over 0.8 based on the predicted and known Morgan fingerprints [37–39]. Using this condition, DeepSAT showed outstanding performance compared to other available tools. Compared to SMART 2.0 (40.3%), the number of Top-1 correct annotations was almost 1.8 fold higher in DeepSAT (70.4%); this reached 73.5% when using the Multiplicity Edited HSQC as input. The result from searching NMRshiftDB by chemical shifts was under 5% for both carbon (3.7%) and proton (1.6%) data. The baseline experiment described above provided 50.6% for Top-1 correct annotations, a higher value than SMART 2.0, but it was still 20% lower than DeepSAT (Fig. 2f).

To compare the annotation performance of DeepSAT with other tools, we varied the top *k* values of the precision, recall, and F1 score to evaluate how each algorithm identifies relevant structures from their predicted features (Table 2). The precision values of the columns *k* = 1, *k* = 5, and *k* = 10 show the number of relevant structures retrieved at the top 1, top 5, and top 10 categories. In the top 1 output, the two versions of DeepSAT achieved 30.0% (HSQC) and 33.5% (Multiplicity Edited HSQC) higher precision values than those of SMART 2.0. The recall values show approximately 5.6% and 6.9% improvements on recall of the top 1 compared with SMART 2.0. The F1 score, which is the harmonic mean of the precision and recall, was also higher in DeepSAT. Accordingly, DeepSAT provided annotations of relevant molecular structures with higher overall similarity scores.

**Table 2:** The precision@k, recall@k, and F1 score@K of structure annotation from different versions of DeepSAT compared with SMART 2.0.

Model	Precision@*k*			Recall@*k*			F1 score@K		
	*k* = *1*	*k* = *5*	*k* = *10*	*k* = *1*	*k* = *5*	*k* = *10*	*k* = *1*	*k* = *5*	*k* = *10*
DeepSAT (multiplicty edited HSQC)	**0.7351**	**0.6283**	**0.5358**	**0.1289**	**0.3917**	**0.5349**	**0.2193**	**0.4826**	**0.5353**
DeepSAT (normal HSQC)	0.7037	0.6056	0.5232	0.1153	0.3585	0.5036	0.1981	0.4504	0.5132
SMART 2.0	0.4032	0.2800	0.2225	0.0596	0.1565	0.2131	0.1038	0.2008	0.2177

Best values in each column are bolded

Another issue to consider in the identification/annotation of molecules from NMR data is that chemical shifts can be altered by a change in solvent, and especially between protic/aprotic solvent conditions. To explore this in the context of DeepSAT, we evaluated its performance under different solvent conditions. We acquired the 78 HSQC spectra for small molecules dissolved in methanol-*d*_4_ (n = 39) or chloroform-*d* (n = 39), respectively; among these, 18 compounds were dissolved in both solvents. As expected, the chemical shifts of the same compound were different in the two solvents (Fig. 2g). For example, several chemical shifts for the diterpene alkaloid neoline were shifted in a nonparallel manner over 0.1 ppm in the proton dimension and 1 ppm in the carbon dimension. The correct identification rate of the top 1 output was 74.4% in chloroform-*d*, and this decreased to 66.7% in methanol-*d*_4_ (Fig. 2h). However, the annotation rates for the top 5 compounds were similar in the two solvents. This trend in the data was also observed for those compounds for which NMR data was recorded in both solvents (Fig. 2i). The increased accuracy in chloroform-*d* is reasonable because most the calculated NMR spectra in the training set were performed in this solvent. Nevertheless, the identification rates were still higher than 65% for the top 1 result, indicating that DeepSAT is still capable of predicting useful structural information in different solvent conditions.

**Structure annotation of previously undescribed natural products**

To illustrate the usefulness of annotations in the molecular structure assignment of previously undescribed molecules of which NMR data have never been reported before, we applied DeepSAT to the HSQC spectra of natural products from a terrestrial medicinal plant (*Agrimonia pilosa*) and a marine brown algae (*Dictyota sp.*). The annotation results for two molecules were compared with the fully assigned structures (i.e. based on ^1^H, ^13^C, COSY, HSQC, HMBC, and NOESY experiments). The molecular structure of compound **1** was annotated by DeepSAT as 3-hydroxy-30-norolean-12,19-dien-28-oic acid and its scaffold was predicted as a triterpenoid (Fig. 2j). However, the reference NMR spectrum from the literature for 3-hydroxy-30-norolean-12,19-dien-28-oic acid did not match that of compound **1** [40]. By detailed NMR analysis, the structure of compound **1** was assigned as 1,2,3-trihydroxyursa-12,18-dien-28-oic acid, a previously undescribed ursane-type triterpenoid. DeepSAT analysis predicted the structure of compound **2** as pulicanadiene C and its scaffold as a diterpenoid (Fig. 2k). By analysis of the full NMR dataset, compound **2** was assigned as 14,15-dihydroxy acutilol A 8-acetate. Interestingly, even though the annotated structure was predicted to be a sequiterpenoid diacetate, the scaffold was correctly predicted as a diterpenoid. These results reveal that using DeepSAT with previously undescribed small molecules can be useful, as DeepSAT gives clues as to scaffolds and structural motifs that can be compared with the data in the literature and accelerate the structure elucidation process.

### Interpretation of convolutional neural networks in DeepSAT

Computer programs or algorithms are typically debugged or error checked using print, assert, or try-catch tools. However, deep neural networks have been criticized as ‘black boxes’, making it difficult to understand the model and how it works. Nevertheless, understanding or explaining how the neural network works is an important aspect of improving its reliability. For this purpose, we evaluated the CNNs used for DeepSAT by visualizing the correlation between NMR signals and substructures. To understand how DeepSAT makes its decision about substructures from NMR spectra, the occlusion sensitivity technique was applied to analyze which parts of an image are most important for a deep network’s prediction [41]. In occlusion sensitivity, some inputs are masked and the changes in results are then correlated with these changes in inputs. Thus, each peak on the HSQC spectrum was sequentially removed and the changes in the predicted results were observed.

**Fig. 3:**
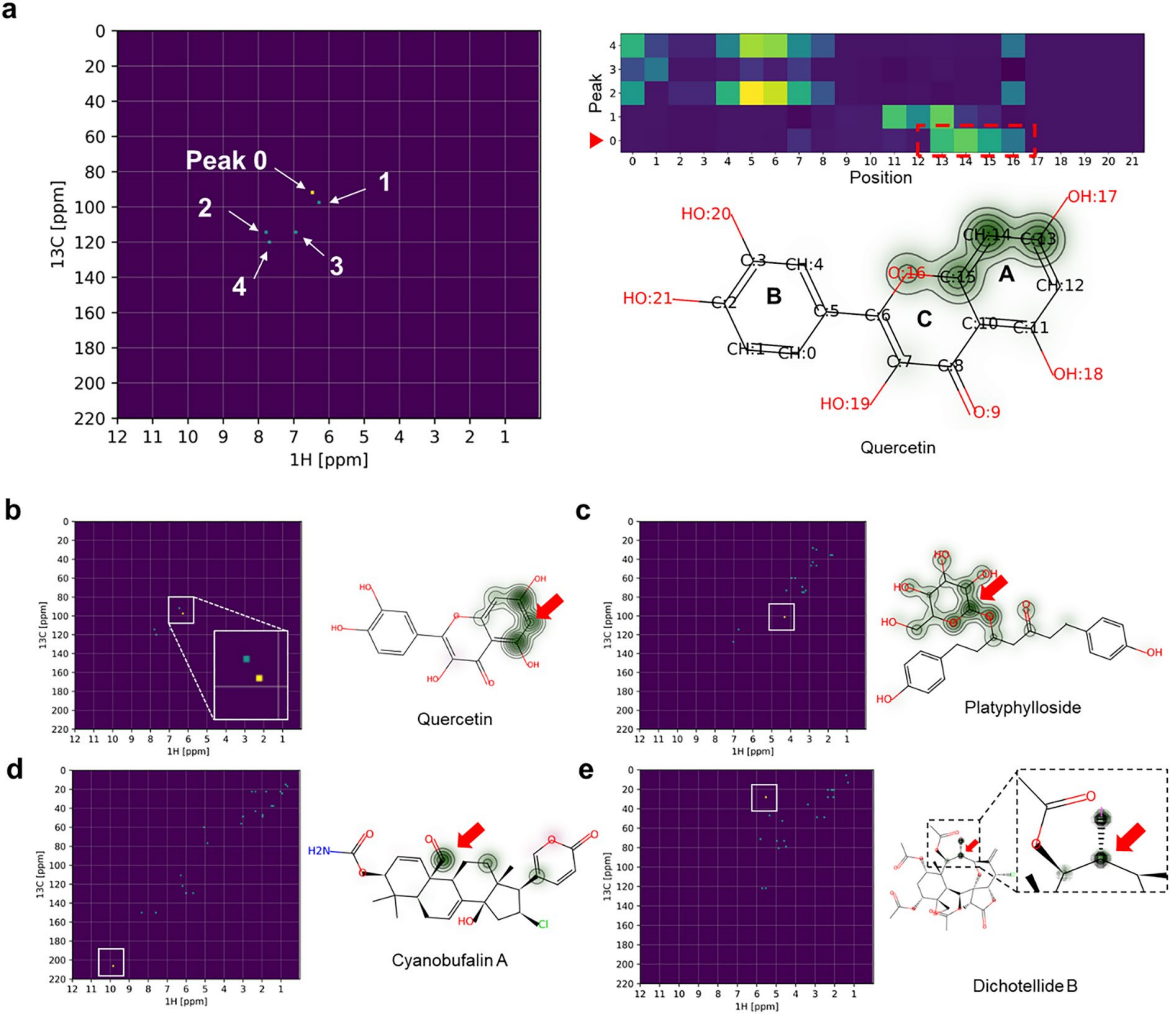
Correlations of HSQC spectra and structural moieties interpreted by the convolutional neural network used by DeepSAT. **a** and **b** HSQC peaks of quercetin are correlated with specific atoms in the molecular structure. The heatmap on the right shows the correlation between the HSQC peaks and atoms on the molecule. Peak 0 was strongly correlated with atom 13,14,15, and 16 (red box). **c–e**, Examples of the correlations interpreted by DeepSAT. The boxed regions of the HSQC spectra on the left are correlated to the functional groups or partial structures on the right and are highlighted by green contour plots. The assigned positions are also marked by red arrows

As a result of this analysis, the change in probability of each atom was mapped onto the source molecule, thereby providing an indication of which substructures are strongly influenced by the selected NMR signals. In Fig. 3, we show the result of this analysis for the compound quercetin. As shown in Figs. 3a, b, the two HSQC peaks at δ_C_/δ_H_ 92.9/6.30 (peak 0) and 97.8/6.19 (peak 1) were significantly correlated with the A ring sub-structure of quercetin. Peak 0 was correlated with C-13, C-14, C-15 and O-16 whereas peak 1 was associated with C-11, C-12 and C-13. Several characteristic correlations between the signals and substructures were observed from this analysis as well. The HSQC signal at δ_C_/δ_H_ 102.0/4.30 was strongly correlated with the anomeric carbon and proton on the glucose moiety of platyphylloside (Fig. 3c). The aldehyde signal at δ_C_/δ_H_ 207.6/9.73 was highly correlated with the aldehyde group on cyanobufalin A (Fig. 3d), and the iodomethylene group of dichotellide B was associated with the signals at δ_C_/δ_H_ 28.7/5.51 (Fig. 3e). Interestingly, these results suggested that the neural network correctly understood the correlation between the functional group and its HSQC signals.

## Discussion

Structure identification from NMR data analysis is an essential process in elucidating the planar and sometimes 3-dimensional structures of organic compounds. Understanding molecular structures provides chemists with the properties of the molecule, which can inform the development of new drugs, the design of new materials, and the understanding of chemical processes in the nature. Thus, the rapid and accurate structure identification and annotation of small molecules by DeepSAT can significantly accelerate such efforts.

DeepSAT resulted in significantly better accuracies than other currently available NMR-based annotation methods. Compared to the next best method, SMART 2.0 or spectral matching with 143,467 reference spectra, DeepSAT showed significantly higher structure identification and annotation rates. Additionally, DeepSAT supports the use of Multiplicity Edited HSQC spectra as inputs, and this provides even higher levels of performance. Because searching and providing the structures from queried spectra is a ‘recommendation system’, we used the appropriate evaluation metrics, including precision, recall, and F1-score @*k* calculated by considering only the subset of the recommended results from rank 1 through *k*. These metrics revealed the significantly improved performance of DeepSAT over SMART 2.0 with a two-fold higher F1-score. These results suggest that DeepSAT produces an excellent prediction of chemical fingerprints and molecular weights as well as structure class prediction from the NMR data.

Because shielding and spin–spin coupling constants in NMR are influenced by the interaction between solvent and solute, the choice in solvent can conceptually influence the results of prediction programs. In evaluating this aspect of the DeepSAT tool, the different solvent conditions were found to only modestly impact the identification and annotation results; over 65% of the results were still correctly identified even though the data were recorded in different solvents, and the annotation rates were even higher. These results indicate that DeepSAT can be a useful identification and annotation tool irrespective of the NMR solvent that is used.

We made initial investigations into how the DeepSAT convolutional neural network recognizes the structural properties of molecules from their NMR spectra using the ‘occlusion sensitivity’ method on the compound quercetin. This analysis provided some basis for understanding how the model recognizes chemical moieties from NMR data. Organic chemists involved in NMR-based structure elucidation commonly correlate chemical shifts and structural motifs based on their experience [42, 43]. Interestingly, this is similar to what was found for DeepSAT. Based on the training of the neural network with large datasets of HSQC spectra, functional groups and structural moieties of small molecules were correctly correlated to their NMR signals. Even though these results do not fully explain the decision-making process of DeepSAT, they suggest that the trained CNN uses empirical correlations similar to those used by human researchers.

## Conclusion

In this study, we introduce DeepSAT, a new tool for the identification and annotation of small molecules using a convolutional deep neural network. DeepSAT possesses a novel architecture in that DeepSAT learned to recognize features of a molecule from HSQC spectra that could be used to populate fingerprint bit strings. As these fingerprints can also be easily generated from all known small molecules to which DeepSAT results can be compared for similarity, it essentially overcomes the data limitation issue present in SMART 1.0, 2.0 and other tools created for this purpose. Furthermore, DeepSAT predicts compound class information for both known and unknown compounds which provides additional insights that are useful in determining molecular structures. In order to validate the performance of DeepSAT, we provided a number of examples of its use, and evaluated its performance using appropriate metrics. The results demonstrate that DeepSAT provides not only structure identification and annotation, but also provides accurate information on the type of small molecules. This state-of-the-art tool for expanding the use of NMR data outperforms all other available tools for identification/annotation of small molecules, and provides multiple types of information that support the structure elucidation process. We anticipate that DeepSAT will be widely used to investigate small molecules for drug discovery applications as well as in ecology and environmental studies.

### Supplementary Information

**Additional file 1: Figure S1.** Input data window from DeepSAT. **Figure S2.** Analysis results from DeepSAT. **Figure S3.** Opening HSQC data file with MestreNova. **Figure S4.** Processing HSQC spectrum. **Figure S5.** Peak picking from HSQC spectrum. **Figure S6.** Run DeepSAT directly on the webpage. **Figure S7.** NMR table format for running DeepSAT. **Figure S8.** NMR table format for diastereotopic protons. **Figure S9.** Copy and paste the peak lists directly from Excel sheets. **Figure S10.** Chemical diversity of molecular structures from reposited NMR spectra in the NMRShiftDB (n=44,315), HMDB (n = 4036), CH-NMR-NP (n = 35,500) by comparing with Dictionary of Natural Products. **Figure S11.** Three constructed HSQC spectra images with different resolutions and the original HSQC spectrum. **Figure S12.** Total loss and performance metrics from training with different image resolutions. **Figure S13.** HSQC spectra computation workflow (left) and its text-formatted output (right). **Figure S14.** Total loss and performance metrics from the training with/without computed HSQC spectra. Validation data was prepared the from literature data and the same in both experiments. **Figure S15.** Confusion matrix of classification results using DeepSAT with normal HSQC data. **Figure S16.** Confusion matrix of classification results using DeepSAT with multiplicity edited HSQC data. **Figure S17.** The precision@k, recall@k, and F1 score@K of structure annotation from different versions of SMART. **Figure S18.** Top1 results from DeepSAT analysis in methanol-*d*_4_ and chloroform-*d*. **Table S1.** Hyperparameters for training the deep neural networks for DeepSAT. **Table S2.** Precision, recall and F1-Score of class prediction results from normal HSQC data. **Table S3.** Precision, recall and F1-Score of class prediction results from Multiplicity-HSQC data.

## Data Availability

The datasets used to train and evaluate DeepSAT are available on GitHub at https://github.com/mwang87/DeepSAT. The Python code used to implement DeepSAT is available on GitHub at https://github.com/mwang87/DeepSAT. DeepSAT is available under the MIT License.
