# Supplementary material for: DeepSAT: Learning Molecular Structures from Nuclear Magnetic Resonance Data
Source: J Cheminform. 2023 Aug 7;15:71. doi: 10.1186/s13321-023-00738-4 (PMC10406729; doi:10.1186/s13321-023-00738-4)
Supplement: Supplementary file 1 — Additional file 1: Figure S1. Input data window from DeepSAT. Figure S2. Analysis results from DeepSAT. Figure S3. Opening HSQC data file with MestreNova. Figure S4. Processing HSQC spectrum. Figure S5. Peak picking from HSQC spectrum. Figure S6. Run DeepSAT directly on the webpage. Figure S7. NMR table format for running DeepSAT. Figure S8. NMR table format for diastereotopic protons. Figure S9. Copy and paste the peak lists directly from Excel sheets. Figure S10. Chemical diversity of molecular structures from reposited NMR spectra in the NMRShiftDB (n=44,315), HMDB (n = 4036), CH-NMR-NP (n = 35,500) by comparing with Dictionary of Natural Products. Figure S11. Three constructed HSQC spectra images with different resolutions and the original HSQC spectrum. Figure S12. Total loss and performance metrics from training with different image resolutions. Figure S13. HSQC spectra computation workflow (left) and its text-formatted output (right). Figure S14. Total loss and performance metrics from the training with/without computed HSQC spectra. Validation data was prepared the from literature data and the same in both experiments. Figure S15. Confusion matrix of classification results using DeepSAT with normal HSQC data. Figure S16. Confusion matrix of classification results using DeepSAT with multiplicity edited HSQC data. Figure S17. The precision@k, recall@k, and F1 score@K of structure annotation from different versions of SMART. Figure S18. Top1 results from DeepSAT analysis in methanol-d4 and chloroform-d. Table S1. Hyperparameters for training the deep neural networks for DeepSAT. Table S2. Precision, recall and F1-Score of class prediction results from normal HSQC data. Table S3. Precision, recall and F1-Score of class prediction results from Multiplicity-HSQC data. [file 13321_2023_738_MOESM1_ESM.docx]

**Additional file**

**DeepSAT: Learning Molecular Structures from Nuclear Magnetic Resonance Data**

Hyun Woo Kim^1,2^, Chen Zhang^1,3^, Raphael Reher^1,4^, Mingxun Wang^5,6,7^, Kelsey L Alexander^1,8^, Louis-Félix Nothias^9^, Yoo Kyong Han^10^, Hyeji Shin^10^, Ki Yong Lee^1,10^, Kyu Hyeong Lee^2^, Myeong Ji Kim^2^, Pieter C. Dorrestein^5^, William H Gerwick^*1,5^ and Garrison W Cottrell^*3^

^1^Center for Marine Biotechnology and Biomedicine, Scripps Institution of Oceanography, University of California San Diego, La Jolla, CA, USA.

^2^College of Pharmacy and Integrated Research Institute for Drug Development, Dongguk University-Seoul, Gyeonggi-do, Republic of Korea

^3^Department of Computer Science and Engineering, University of California, San Diego, La Jolla, CA, USA.

^4^Institute of Pharmaceutical Biology and Biotechnology, University of Marburg, Marburg, Germany.

^5^Skaggs School of Pharmacy and Pharmaceutical Sciences, University of California San Diego, La Jolla, CA, USA.

^6^Ometa Labs LLC, San Diego, CA, USA.

^7^Department of Computer Science, University of California Riverside, Riverside, CA, USA

^8^Department of Chemistry and Biochemistry, University of California San Diego, La Jolla, CA, USA.

^9^Institut de Chimie de Nice, Université Côte d’Azur, UMR 7272 CNRS, 06108, Nice, France.

^10^College of Pharmacy, Korea University, Sejong, Republic of Korea.

*Co-corresponding authors.

**Table of contents**

**1. Introduction of the DeepSAT website**

**2. Chemical diversity of publicly available NMR databases**

**3. Constructed HSQC from experimental spectra**

**4. Model training optimization**

**5. Compound class prediction results**

**6. Precision, recall and F1 scores @K of DeepSAT analysis**

**7. Top1 annotation results of the molecules from DeepSAT analysis in different solvent condition**

**8. Compound list of experimental HSQC spectra**

**9. Experimental HSQC spectra in CDCl3 and MeOD**

**10. Structure elucidation of previously unreported compounds from *A. pilosa* and *Dictyota sp.***

**1. Introduction of DeepSAT webpage**

**1.1. Data input**

DeepSAT webpage (<https://deepsat.ucsd.edu>) was designed to provide easy-to-use user interface for structure annotation. The input format is in the form of an NMR peak list; comma-separated or tab-separated formats are supported. After the NMR peak list is input, the experimental type of HSQC needs to be selected between normal HSQC and Multiplicity HSQC. The molecular weight (optional) can be input if users know the molecular weight of the compound. In this case, the input molecular weight is used for annotation instead of the predicted one.


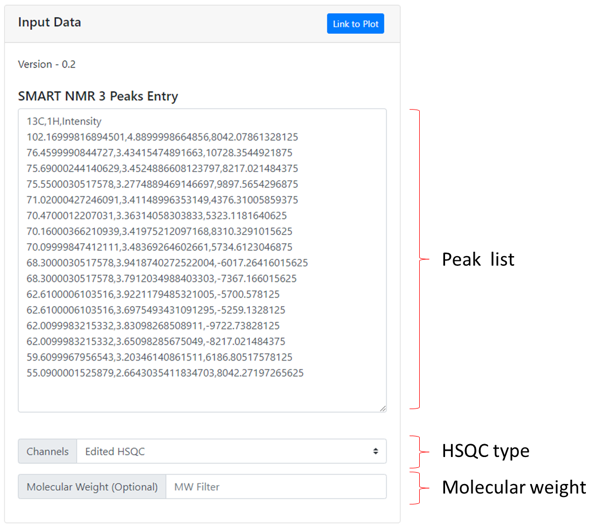


Figure S1 Input data window from DeepSAT

**1.2. Analysis and results**

Once the HSQC peak list, HSQC type and molecular weight (optional) are submitted, the DeepSAT analysis is automatically processed, and results are provided with compound class, compound names, molecular weight, cosine score, and molecular structures.


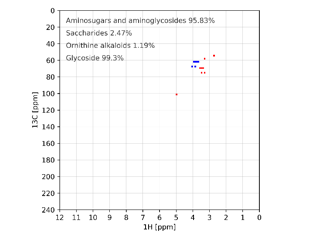


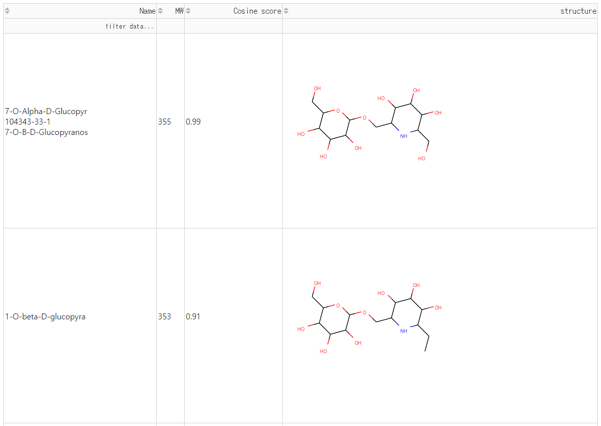


Figure S2 Analysis results from DeepSAT

**1.3. User's Guide for DeepSAT Analysis**

- The current version of DeepSAT as of 05/16/2021 consists of 2D NMR spectra from 144,254 natural products.

- One DeepSAT analysis should take < 20 seconds.

- If your results are unsatisfactory, please try to process your data again manually (go to How to process a raw HSQC spectrum to an NMR table and then delete noise and duplicate annotations, add peaks missed by auto-peak picking etc.)

**1.3.1. How to process a raw HSQC spectrum to an NMR table with MestreNova**

1. Open your raw HSQC spectrum in MestreNova (preferences: modern view)

- Drag&Drop your HSQC file (for Bruker data you find your spectrum under: pdata/1/2rr)

- Depending on purity and concentration of your sample and acquisition time, your spectrum will look more or less clean, and may need additional processing (see 2.)


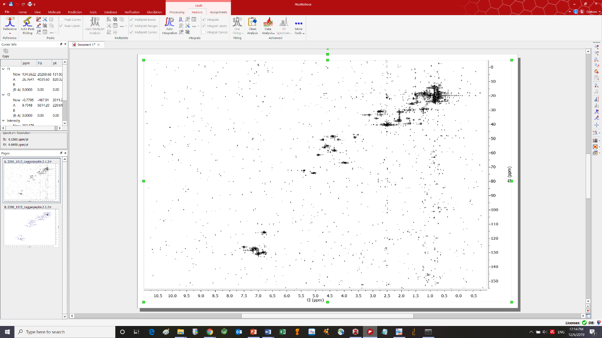


Figure S3 Opening HSQC data file with MestreNova

2. For processing your HSQC spectrum click on 'Processing' tab

- click on 'Auto Phase Correction' (optional: correct manually)

- click on 'Auto Baseline Correction'

- click on 'More Processing' --> click on 'Reduce t1 noise' You should see a clean spectrum now.


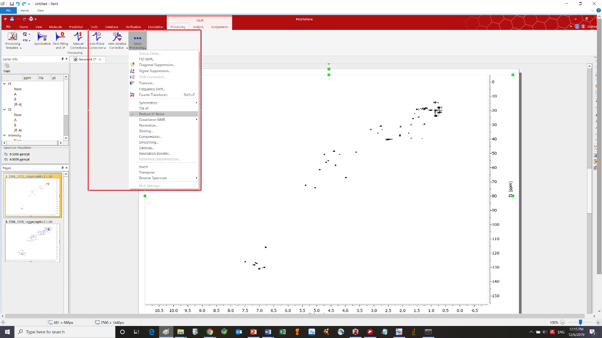


Figure S4 Processing HSQC spectrum

3. Annotate HSQC spectrum with chemical shifts (1H,13C)

- click on 'Analysis' tab

- click on Auto Peak Picking (Important: Check by manually adding missed peaks and removing duplicated, nonsense and solvent peak annotations). Now each peak should be annotated with two numbers separated by comma (1H, 13C chemical shifts)


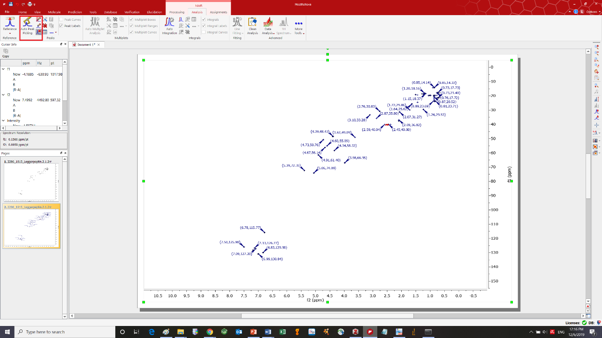


Figure S5 Peak picking from HSQC spectrum

4. Generate NMR table from annotated HSQC spectrum

- click on 'Analysis' tab

- click on 'NMR Peaks Table'

- right click on table, setup report, setup table

- customize table by unchecking every value except f2 (change visible name to 1H), f1 (change visible name to 13C), and Intensity (optional for normal HSQC, essential for Multiplicity-HSQC)

- copy all (ctrl+A)

- click on 'copy peaks' and choose 'copy table'

5. Run SMART Analysis directly

- copy and paste the table (ctrl+C,ctrl+V) directly to the peak list section of https://deepsat.ucsd.edu

- Important: Apply one backslash to remove the additional space character that is imported with the NMR table from MestreNova.

- Select experiment type (Normal HSQC or Multiplicity HSQC)

- (Optional) If you know the molecular weight of your compounds, please enter it.

- Once the data and experimental condition are submitted, analysis is automatically processed.


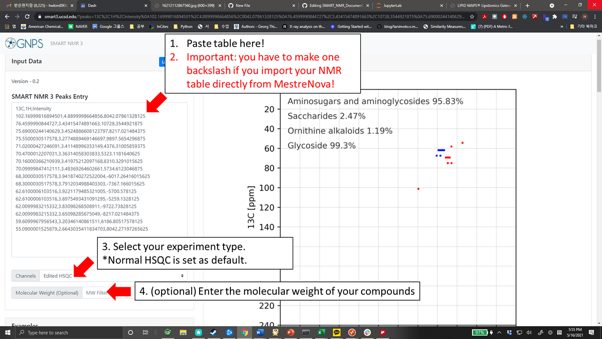


Figure S6 Run DeepSAT directly on the webpage

6. The results will be provided from DeepSAT Analysis on the right side

- Please feel free to play around with the processing parameters such as including/excluding noise signals or signals from other minor compounds in case of mixtures or explore the differences of SMART results when referencing your spectra compared to tables without referencing. Overall SMART is designed to be very robust towards any of these changes as its training is not only based on the absolute position of the peaks, but the relative position of each peak towards every other peak.

**1.3.2. How to process a raw HSQC spectrum to an NMR table with Excel.**

- Please prepare your NMR peak lists of each compound using Excel or preferably notepad/wordpad. The first row will be left for strings “1H”, “13C”, and "Intensity" (optional) as table head (The order of header is nothing to do with the analysis results).


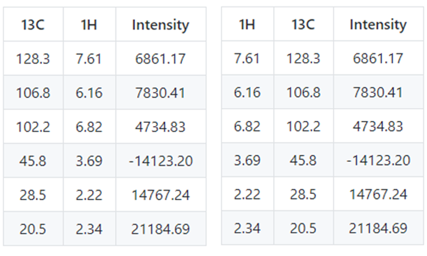


Figure S7 NMR table format for running DeepSAT

- In the NMR table files, wherever there are diastereotopic protons on a methylene carbon (i.e., CH2 with two distinct proton shifts), please add a separate entry for both the carbon and proton:


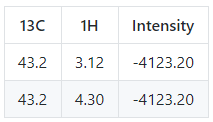


Figure S8 NMR table format for diastereotopic protons

**1.3.3. How to process a raw HSQC spectrum to a NMR table with Notepad/wordpad**

- SMART supports comma-separated values and tap-separated values for analysis. Your table should appear like this:

for comma-separated values:

1H,13C,Intensity

1.09,14.3,132

2.21,22.2,155

3.41,56.9,239

7.21,128.6,443

7.29,123.4,563

or for tap-separated values:

1H 13C Intensity

1.09 14.3 132

2.21 22.2 155

3.41 56.9 239

7.21 128.6 443

7.29 123.4 563

**1.3.4. Copy peak lists from Excel sheets for SMART Analysis**

- If you save or prepare your peak lists with Excel files, the data are easily submitted to DeepSAT by copying and pasting the table.


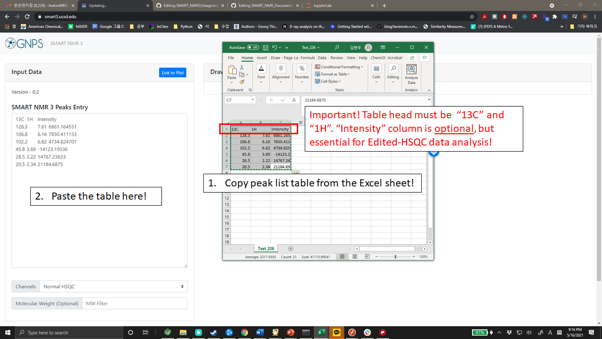


Figure S9 Copy and paste the peak lists directly from Excel sheets.

**2. Chemical diversity of publicly available NMR databases using uniform manifold approximation and projection (UMAP)**


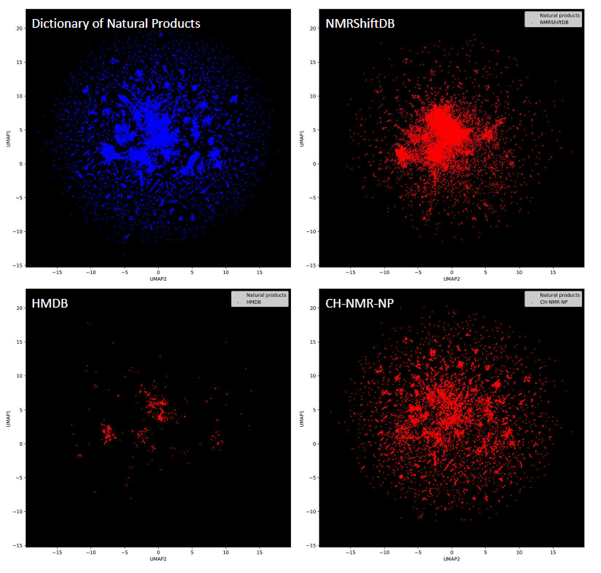


Figure S10 Chemical diversity of molecular structures from reposited NMR spectra in the NMRShiftDB (n=44,315), HMDB (n = 4,036), CH-NMR-NP (n = 35,500) by comparing with Dictionary of Natural Products.

**3. Constructed HSQC from experimental spectra**

**
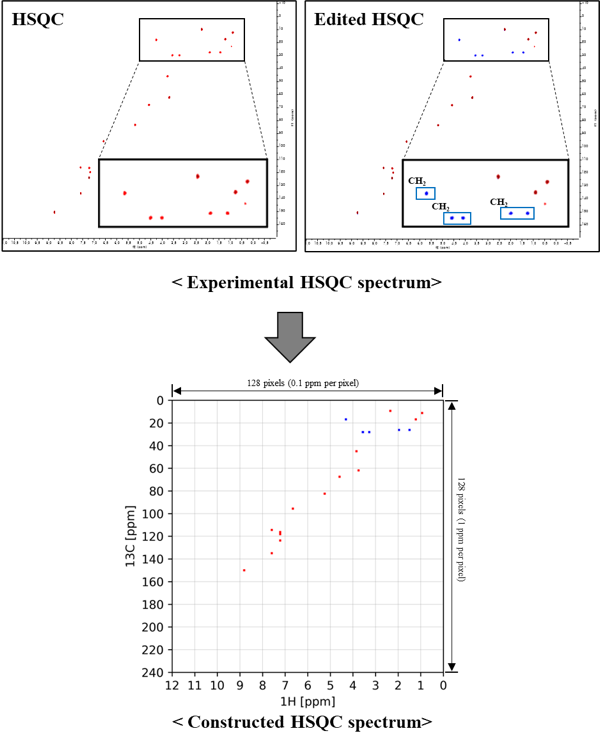
**

**4. Model training optimization**

**4.1. Image resolution for the convolutional neural network**

In order to choose the best image resolution for the convolutional neural network training, three resolutions (64 × 64, 128 × 128, and 192 × 192) of image were tested. Among them, the training from 128 × 128 image resolution showed the best results in the fingerprint and molecular weight predictions.


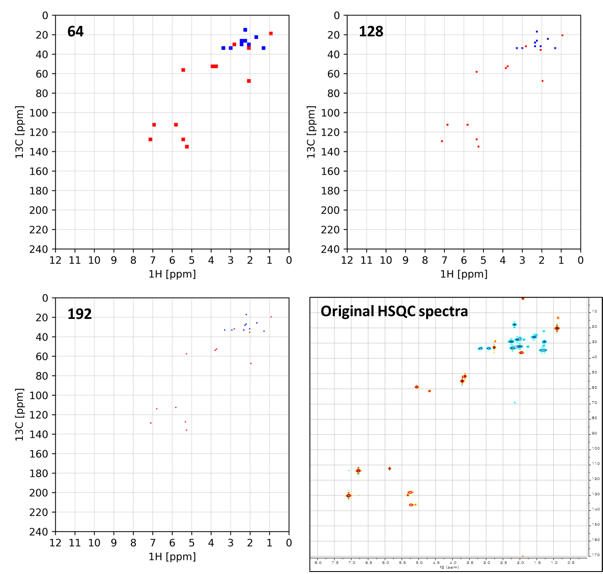


Figure S11 Three constructed HSQC spectra images with different resolutions and the original HSQC spectrum.

**
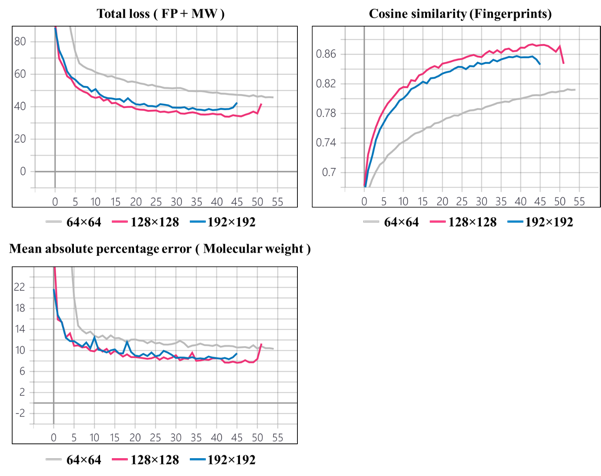
**

Figure S12 Total loss and performance metrics from training with different image resolutions.

**4.2. Data sampling from computed HSQC data for dataset expansion**

**4.2.1 HSQC spectra computation by using the ACD/Spectrus Processor program**


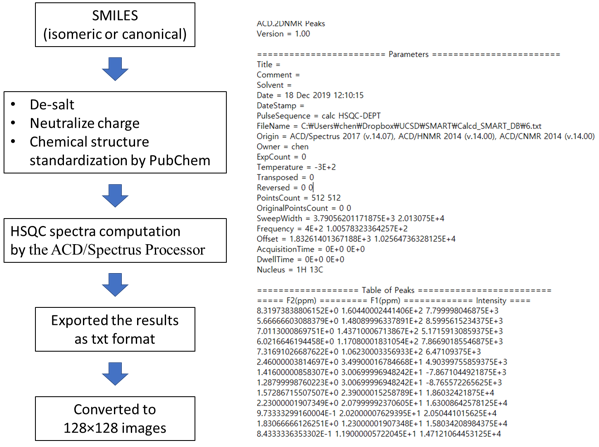


Figure S13 HSQC spectra computation workflow (left) and its text-formatted output (right)

**4.2.2 Confirmation of performance improvement in the training by using computed HSQC spectra.**

To confirm that the training set with computed HSQC data improved performance, the training results from two datasets with and without computed HSQC data were compared to each other. As we expected, the expanded dataset with computed HSQC data showed the best training results.


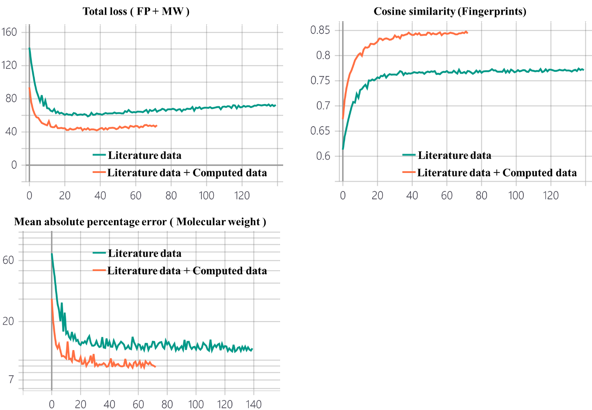


Figure S14 Total loss and performance metrics from the training with/without computed HSQC spectra. Validation data was prepared the from literature data and the same in both experiments.

Table S1 Hyperparameters for training the deep neural networks for DeepSAT

| Optimizer | Adam |
| --- | --- |
| Activation functions | ReLu (hidden layers) Sigmoid (fingerprint prediction layer)  Softmax (classification layer) |
| Loss function | Binary cross-entropy (fingerprint prediction) Sparse categorical cross entropy (classification) Mean absolute percentage error (molecular weight regression) |
| Regularization | L2 regularizer (0.0001, fully connected layer) |
| Learning rate | 0.00001 |
| Batch normalization | Used |
| Dropout rate | 0.2 |
| Batch size | 16 |

**5. Compound class prediction results**

Table S2 Precision, recall and F1-Score of class prediction results from normal HSQC data. For the purposes of this model, we ignored the 11 superclasses in NPClassifier that were not represented in our data. All the results reported here are based on a 59-way classification.

| **No.** | **Class** | **Precision** | **Recall** | **F1-score** |
| --- | --- | --- | --- | --- |
| 1 | Amino acid glycosides | 0.867 | 0.722 | 0.788 |
| 2 | Aminosugars and aminoglycosides | 1.000 | 0.565 | 0.722 |
| 3 | Anthranilic acid alkaloids | 0.713 | 0.713 | 0.713 |
| 4 | Apocarotenoids | 0.770 | 0.851 | 0.809 |
| 5 | Aromatic polyketides | 0.799 | 0.840 | 0.819 |
| 6 | Carotenoids (C40) | 0.990 | 0.910 | 0.948 |
| 7 | Chromanes | 0.912 | 0.752 | 0.824 |
| 8 | Coumarins | 0.739 | 0.889 | 0.807 |
| 9 | Cyclic polyketides | 0.899 | 0.738 | 0.811 |
| 10 | Diarylheptanoids | 0.912 | 0.881 | 0.897 |
| 11 | Diazotetronic acids and derivatives | 0.800 | 0.533 | 0.640 |
| 12 | Diphenyl ethers (DPEs) | 0.727 | 0.800 | 0.762 |
| 13 | Diterpenoids | 0.949 | 0.938 | 0.943 |
| 14 | Eicosanoids | 1.000 | 0.973 | 0.986 |
| 15 | Fatty Acids and Conjugates | 0.830 | 0.824 | 0.827 |
| 16 | Fatty acyl glycosides | 0.917 | 0.917 | 0.917 |
| 17 | Fatty acyls | 0.855 | 0.851 | 0.853 |
| 18 | Fatty amides | 0.946 | 0.778 | 0.854 |
| 19 | Fatty esters | 0.830 | 0.557 | 0.667 |
| 20 | Flavonoids | 0.918 | 0.925 | 0.922 |
| 21 | Glycerolipids | 0.941 | 0.979 | 0.960 |
| 22 | Glycerophospholipids | 0.930 | 1.000 | 0.964 |
| 23 | Guanidine alkaloids | 0.842 | 0.800 | 0.821 |
| 24 | Histidine alkaloids | 0.818 | 0.450 | 0.581 |
| 25 | Isoflavonoids | 0.948 | 0.932 | 0.940 |
| 26 | Lignans | 0.965 | 0.918 | 0.941 |
| 27 | Linear polyketides | 0.963 | 0.892 | 0.926 |
| 28 | Lysine alkaloids | 0.737 | 0.828 | 0.780 |
| 29 | Macrolides | 0.980 | 0.943 | 0.961 |
| 30 | Meroterpenoids | 0.775 | 0.855 | 0.813 |
| 31 | Monoterpenoids | 0.907 | 0.822 | 0.862 |
| 32 | Naphthalenes | 0.879 | 0.533 | 0.664 |
| 33 | Nicotinic acid alkaloids | 0.916 | 0.731 | 0.813 |
| 34 | Nucleosides | 0.983 | 1.000 | 0.991 |
| 35 | Oligopeptides | 0.975 | 0.994 | 0.984 |
| 36 | Ornithine alkaloids | 0.894 | 0.789 | 0.838 |
| 37 | Phenolic acids (C6-C1) | 0.780 | 0.773 | 0.776 |
| 38 | Phenylethanoids (C6-C2) | 0.941 | 0.941 | 0.941 |
| 39 | Phenylpropanoids (C6-C3) | 0.750 | 0.926 | 0.829 |
| 40 | Phloroglucinols | 0.932 | 0.885 | 0.908 |
| 41 | Polycyclic aromatic polyketides | 0.807 | 0.888 | 0.846 |
| 42 | Polyethers | 0.884 | 1.000 | 0.938 |
| 43 | Polyols | 0.800 | 0.632 | 0.706 |
| 44 | Proline alkaloids | 0.750 | 0.643 | 0.692 |
| 45 | Pseudoalkaloids (transamidation) | 0.840 | 0.782 | 0.810 |
| 46 | Saccharides | 0.818 | 0.931 | 0.871 |
| 47 | Sesquiterpenoids | 0.866 | 0.935 | 0.899 |
| 48 | Sesterterpenoids | 0.946 | 0.921 | 0.933 |
| 49 | Small peptides | 0.758 | 0.851 | 0.802 |
| 50 | Spingolipids | 0.933 | 0.990 | 0.960 |
| 51 | Steroids | 0.962 | 0.974 | 0.968 |
| 52 | Stilbenoids | 0.921 | 0.894 | 0.907 |
| 53 | Styrylpyrones | 0.905 | 1.000 | 0.950 |
| 54 | Terphenyls | 0.821 | 0.793 | 0.807 |
| 55 | Triterpenoids | 0.979 | 0.972 | 0.975 |
| 56 | Tryptophan alkaloids | 0.853 | 0.955 | 0.901 |
| 57 | Tyrosine alkaloids | 0.954 | 0.903 | 0.928 |
| 58 | Xanthones | 0.892 | 0.734 | 0.805 |
| 59 | beta-lactams | 1.000 | 1.000 | 1.000 |


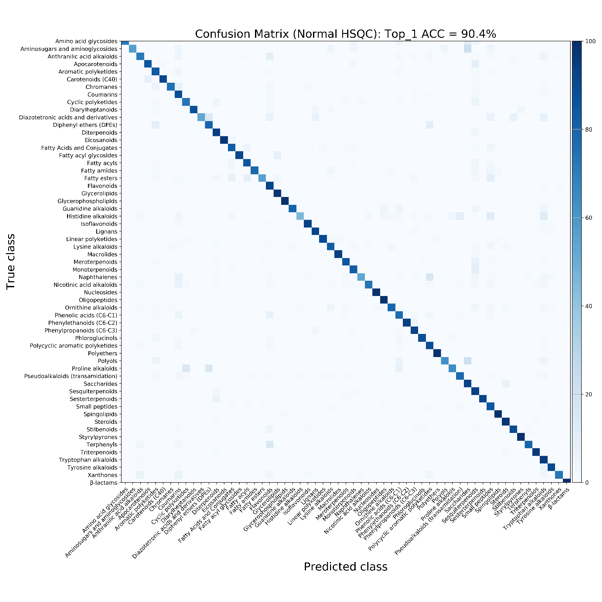


Figure S15 Confusion matrix of classification results using DeepSAT with normal HSQC data.

Table S3. Precision, recall and F1-Score of class prediction results from multiplicity edited HSQC data. For the purposes of this model, we ignored the 11 superclasses in NPClassifier that were not represented in our data. All the results reported here are based on a 59-way classification.

| **No.** | **Class** | **Precision** | **Recall** | **F1-score** |
| --- | --- | --- | --- | --- |
| 1 | Amino acid glycosides | 0.682 | 0.833 | 0.750 |
| 2 | Aminosugars and aminoglycosides | 0.773 | 0.739 | 0.756 |
| 3 | Anthranilic acid alkaloids | 0.646 | 0.689 | 0.667 |
| 4 | Apocarotenoids | 0.612 | 0.896 | 0.727 |
| 5 | Aromatic polyketides | 0.875 | 0.808 | 0.840 |
| 6 | Carotenoids (C40) | 0.981 | 0.937 | 0.959 |
| 7 | Chromanes | 0.763 | 0.847 | 0.803 |
| 8 | Coumarins | 0.915 | 0.826 | 0.868 |
| 9 | Cyclic polyketides | 0.820 | 0.786 | 0.803 |
| 10 | Diarylheptanoids | 0.926 | 0.847 | 0.885 |
| 11 | Diazotetronic acids and derivatives | 0.667 | 0.400 | 0.500 |
| 12 | Diphenyl ethers (DPEs) | 0.765 | 0.650 | 0.703 |
| 13 | Diterpenoids | 0.945 | 0.962 | 0.953 |
| 14 | Eicosanoids | 0.974 | 1.000 | 0.987 |
| 15 | Fatty Acids and Conjugates | 0.771 | 0.865 | 0.815 |
| 16 | Fatty acyl glycosides | 0.900 | 0.750 | 0.818 |
| 17 | Fatty acyls | 0.695 | 0.931 | 0.796 |
| 18 | Fatty amides | 0.946 | 0.778 | 0.854 |
| 19 | Fatty esters | 0.822 | 0.759 | 0.789 |
| 20 | Flavonoids | 0.892 | 0.932 | 0.912 |
| 21 | Glycerolipids | 0.979 | 0.969 | 0.974 |
| 22 | Glycerophospholipids | 0.907 | 0.975 | 0.940 |
| 23 | Guanidine alkaloids | 1.000 | 0.750 | 0.857 |
| 24 | Histidine alkaloids | 0.700 | 0.700 | 0.700 |
| 25 | Isoflavonoids | 0.967 | 0.885 | 0.924 |
| 26 | Lignans | 0.957 | 0.948 | 0.953 |
| 27 | Linear polyketides | 0.920 | 0.944 | 0.932 |
| 28 | Lysine alkaloids | 0.742 | 0.779 | 0.760 |
| 29 | Macrolides | 0.980 | 0.943 | 0.961 |
| 30 | Meroterpenoids | 0.888 | 0.812 | 0.848 |
| 31 | Monoterpenoids | 0.888 | 0.845 | 0.866 |
| 32 | Naphthalenes | 0.719 | 0.700 | 0.709 |
| 33 | Nicotinic acid alkaloids | 0.819 | 0.827 | 0.823 |
| 34 | Nucleosides | 0.982 | 0.931 | 0.956 |
| 35 | Oligopeptides | 0.977 | 0.983 | 0.980 |
| 36 | Ornithine alkaloids | 0.745 | 0.867 | 0.801 |
| 37 | Phenolic acids (C6-C1) | 0.730 | 0.836 | 0.780 |
| 38 | Phenylethanoids (C6-C2) | 1.000 | 0.882 | 0.938 |
| 39 | Phenylpropanoids (C6-C3) | 0.798 | 0.926 | 0.857 |
| 40 | Phloroglucinols | 0.938 | 0.769 | 0.845 |
| 41 | Polycyclic aromatic polyketides | 0.849 | 0.867 | 0.858 |
| 42 | Polyethers | 1.000 | 0.974 | 0.987 |
| 43 | Polyols | 0.867 | 0.684 | 0.765 |
| 44 | Proline alkaloids | 0.889 | 0.571 | 0.696 |
| 45 | Pseudoalkaloids (transamidation) | 0.952 | 0.690 | 0.800 |
| 46 | Saccharides | 0.957 | 0.776 | 0.857 |
| 47 | Sesquiterpenoids | 0.939 | 0.915 | 0.926 |
| 48 | Sesterterpenoids | 0.989 | 0.816 | 0.894 |
| 49 | Small peptides | 0.951 | 0.703 | 0.808 |
| 50 | Spingolipids | 0.942 | 0.990 | 0.965 |
| 51 | Steroids | 0.980 | 0.988 | 0.984 |
| 52 | Stilbenoids | 0.922 | 0.904 | 0.913 |
| 53 | Styrylpyrones | 0.929 | 0.684 | 0.788 |
| 54 | Terphenyls | 0.647 | 0.759 | 0.698 |
| 55 | Triterpenoids | 0.975 | 0.985 | 0.980 |
| 56 | Tryptophan alkaloids | 0.931 | 0.907 | 0.919 |
| 57 | Tyrosine alkaloids | 0.911 | 0.957 | 0.933 |
| 58 | Xanthones | 0.687 | 0.815 | 0.745 |
| 59 | beta-lactams | 1.000 | 0.950 | 0.974 |

**
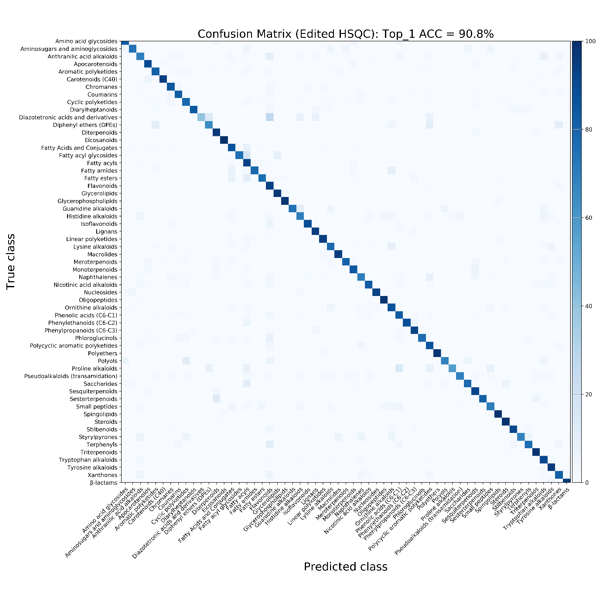
**

Figure S16 Confusion matrix of classification results using DeepSAT with multiplicity edited HSQC data.

**6. Precision, recall and F1 scores @K of structure annotation results from DeepSAT**


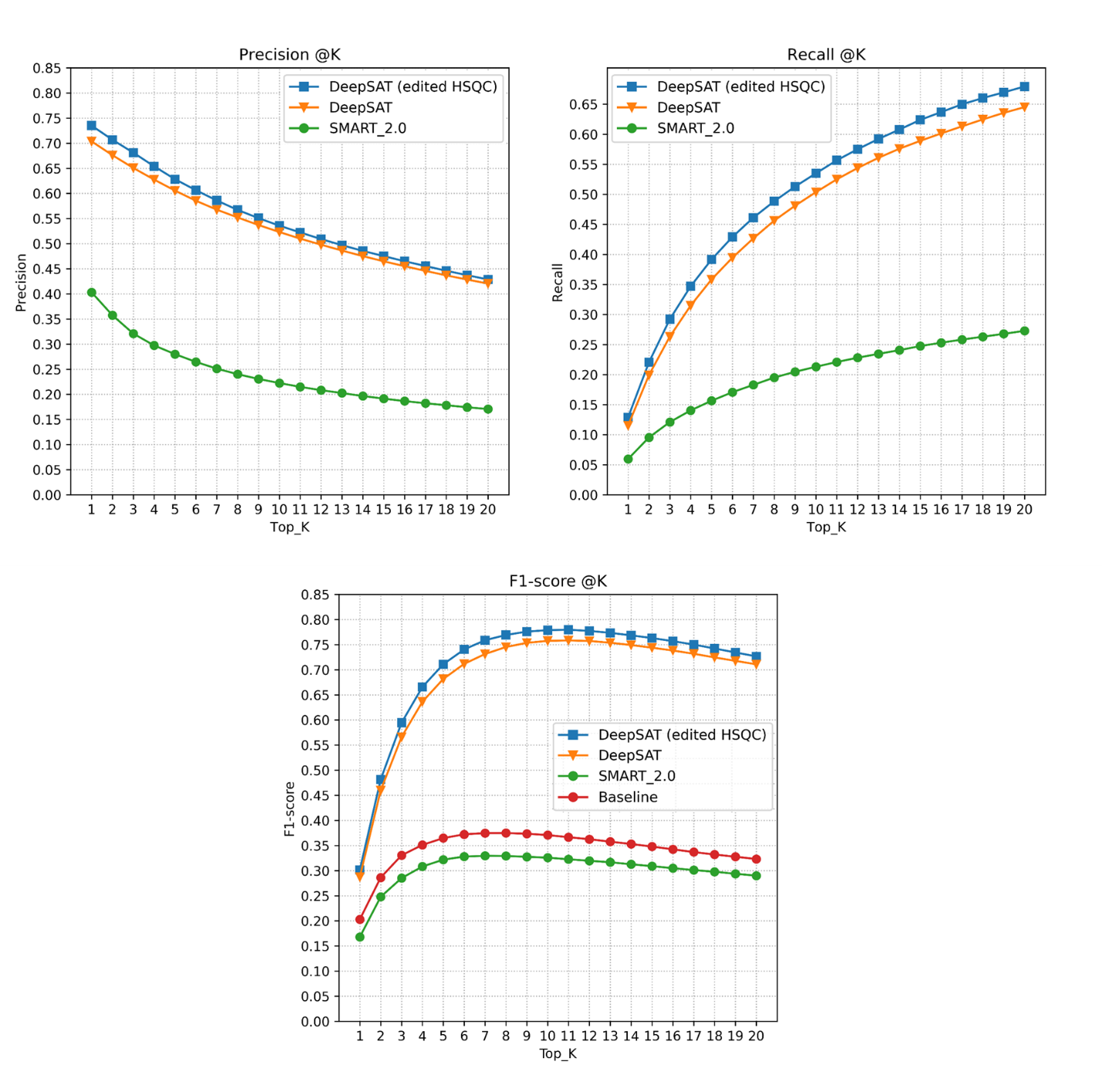


Figure S17 The precision@k, recall@k, and F1 score@K of structure annotation from DeepSAT and SMART 2.0

**7.** **Top1 annotation results from DeepSAT analysis in different solvent conditions. (Ground truth, predicted from MeOD, predicted from CDCl_3_)**

***
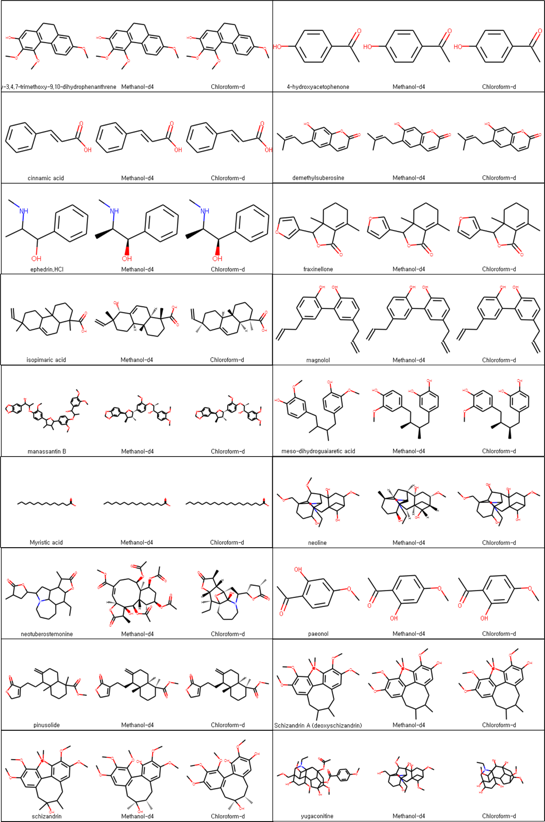
***

Figure S18 Top1 results from DeepSAT analysis in methanol-*d*_4_ and chloroform-*d*

**8. Compound list of the experimental HSQC spectra (“O” indicates that a spectrum was recorded in that solvent, “X” indicates that a spectrum was not recorded in that solvent).**

| **NO.** | **Name** | **Molecular Formula** | **M.W.** | **HSQC data** | |
| --- | --- | --- | --- | --- | --- |
|  |  |  |  | **CDCl3** | **MeOD** |
| 1 | Neoline | C_24_H_39_NO_6_ | 437.6 | **O** | **O** |
| 2 | hypaconitine | C_33_H_45_NO_10_ | 615.7 | **O** | **X** |
| 3 | tugaconitine | C_23_H_35_NO_7_ | 437.5 | **O** | **O** |
| 4 | Decursin | C_19_H_20_O_5_ | 328.4 | **O** | **X** |
| 5 | decursinol | C_14_H_14_O_4_ | 246.3 | **O** | **X** |
| 6 | demethylsuberosin | C_14_H_14_O_3_ | 230.3 | **O** | **X** |
| 7 | isoimperatorin | C_16_H_14_O_4_ | 270.3 | **O** | **X** |
| 8 | isopimaric acid | C_20_H_32_O | 288.5 | **O** | **O** |
| 9 | pinusolide | C_21_H_30_O_4_ | 346.5 | **O** | **O** |
| 10 | Schizandrin A (deoxyschizandrin) | C_24_H_32_O_6_ | 416.5 | **O** | **O** |
| 11 | meso-dihydroguaiaretic acid | C_20_H_26_O_4_ | 330.4 | **O** | **O** |
| 12 | quercetin | C_15_H_10_O_7_ | 302.2 | **X** | **O** |
| 13 | manassantin B | C_41_H_48_O_11_ | 716.8 | **O** | **O** |
| 14 | sauchinone | C_20_H_20_O_6_ | 356.1 | **O** | **X** |
| 15 | gomisin N | C_23_H_28_O_6_ | 400.5 | **O** | **X** |
| 16 | wuweizisu C | C_22_H_24_O_6_ | 384.4 | **O** | **X** |
| 17 | cinnamic acid | C_9_H_8_O_2_ | 148.2 | **O** | **O** |
| 18 | E-p-coumaric acid | C_9_H_8_O_3_ | 164.2 | **X** | **O** |
| 19 | scopolamine | C_17_H_21_NO_4_ | 303.4 | **X** | **O** |
| 20 | diosgenin | C_27_H_42_O_3_ | 414.6 | **O** | **X** |
| 21 | Catechin | C_15_H_14_O_6_ | 290.3 | **X** | **O** |
| 22 | ursodeoxycholic acid | C_24_H_40_O_4_ | 392.6 | **X** | **O** |
| 23 | Loganin | C_17_H_26_O_10_ | 390.3 | **X** | **O** |
| 24 | Puerarin | C_21_H_20_O_9_ | 416.4 | **X** | **O** |
| 25 | schizandrin | C_24_H_32_O_7_ | 432.5 | **O** | **O** |
| 26 | ephedrin.HCl | C_10_H_16_NOCl | 210.1 | **O** | **O** |
| 27 | friedeline | C_30_H_50_O | 426.0 | **O** | **X** |
| 28 | glut-5-enol | C_30_H_50_O | 426.0 | **O** | **X** |
| 29 | a-amyrin | C_30_H_50_O | 426.0 | **O** | **X** |
| 30 | b-amyrin | C_30_H_50_O | 426.0 | **O** | **X** |
| 31 | gallic acid | C_7_H_6_O_5_ | 170.0 | **X** | **O** |
| 32 | eclalbasaponin III | C_48_H_78_O_19_ | 959.1 | **X** | **O** |
| 33 | 4-hydroxyacetophenone | C_8_H_8_O_2_ | 136.1 | **O** | **O** |
| 34 | fraxinellone | C_14_H_16_O_3_ | 232.3 | **O** | **O** |
| 35 | 2-hydroxy-3,4,7-trimethoxy-9,10-dihydrophenanthrene | C_17_H_18_O_4_ | 286.3 | **O** | **O** |
| 36 | erianthridin | C_16_H_16_O_5_ | 272.0 | **X** | **O** |
| 37 | lusianthridin | C_15_H_14_O_3_ | 242.0 | **X** | **O** |
| 38 | gallic acid methyl ester | C_8_H_8_O_5_ | 184.2 | **X** | **O** |
| 39 | nordihydroguaiaretic acid | C_18_H_22_O_4_ | 302.4 | **X** | **O** |
| 40 | magnolol | C_18_H_18_O_2_ | 266.3 | **O** | **O** |
| 41 | geniposide | C_17_H_24_O_10_ | 388.4 | **X** | **O** |
| 42 | Paeonol | C_9_H_10_O_3_ | 166.2 | **O** | **O** |
| 43 | machilin A | C_20_H_22_O_4_ | 326.4 | **O** | **X** |
| 44 | demethylsuberosine | C_14_H_14_O_3_ | 230.3 | **O** | **O** |
| 45 | asiatic acid | C_30_H_48_O_5_ | 488.7 | **X** | **O** |
| 46 | Galbacin | C_20_H_20_O_5_ | 340.4 | **O** | **X** |
| 47 | Lyoniside | C_27_H_36_O_12_ | 552.6 | **X** | **O** |
| 48 | 3-hydroxy-5,7-dimethoxy-3,4-methylenedioxyflavan | C_18_H_18_O_6_ | 330.0 | **O** | **X** |
| 49 | 3,4-dihydroxy-3,5,7-trimethoxyflavan | C_18_H_20_O_6_ | 332.0 | **O** | **X** |
| 50 | azelaic acid | C_9_H_16_O_4_ | 154.0 | **X** | **O** |
| 51 | neotuberostemonine | C_22_H_33_NO_4_ | 375.2 | **O** | **O** |
| 52 | Indoxyl B-D-glucoside (indican) | C_14_H_17_NO_6_ | 295.1 | **X** | **O** |
| 53 | 3,4-dihydroxybenzoic acid | C_7_H_6_O_4_ | 154.1 | **X** | **O** |
| 54 | Myristic acid | C_14_H_28_O_2_ | 228.4 | **O** | **O** |
| 55 | Stearic acid | C_18_H_36_O_2_ | 284.5 | **O** | **X** |
| 56 | platyphylloside | C_25_H_32_O_9_ | 476.5 | **X** | **O** |
| 57 | 3,5,7,2',6'-pentahydroxyflavanone | C_15_H_12_O_7_ | 304.3 | **X** | **O** |
| 58 | imperatorin | C_16_H_14_O_4_ | 270.3 | **O** | **X** |
| 59 | oxypeucedanin | C_16_H_14_O_5_ | 286.3 | **O** | **X** |
| 60 | arctigenin | C_21_H_24_O_6_ | 372.4 | **O** | **X** |

**9. Experimental HSQC spectra in CDCl3 and MeOD**

**9.1. Experimental HSQC spectra in CDCl_3_**


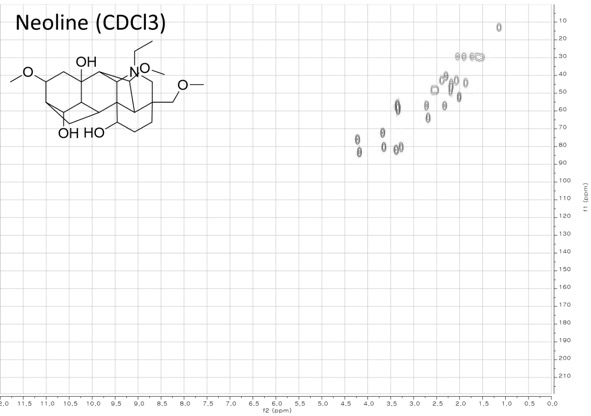


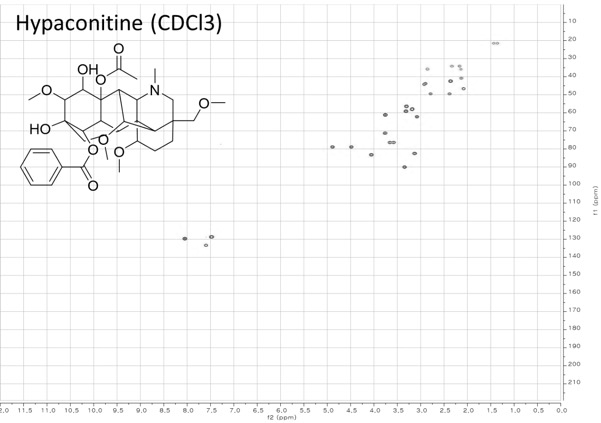


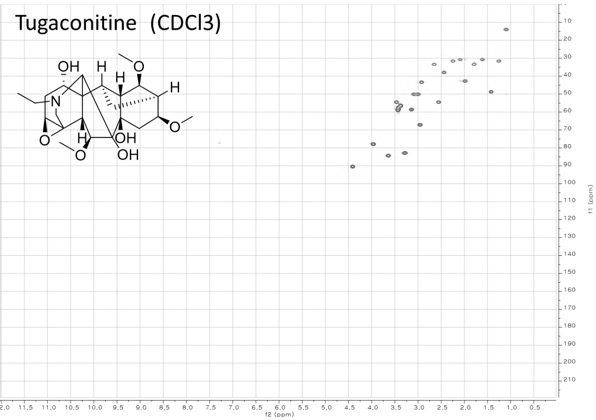


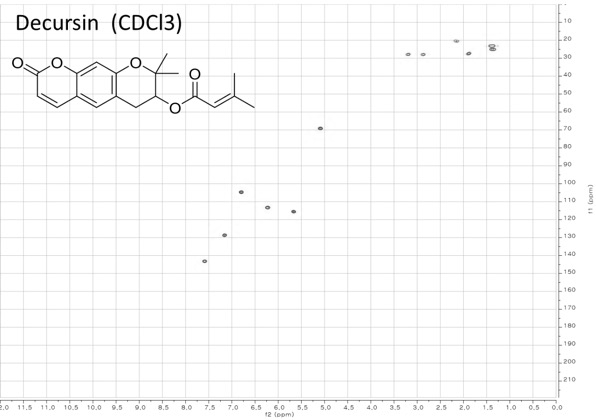


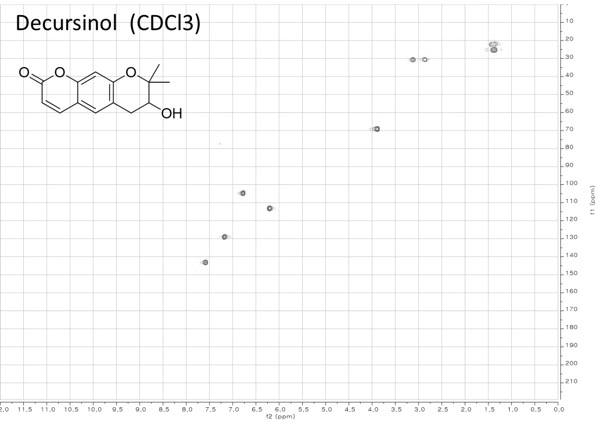


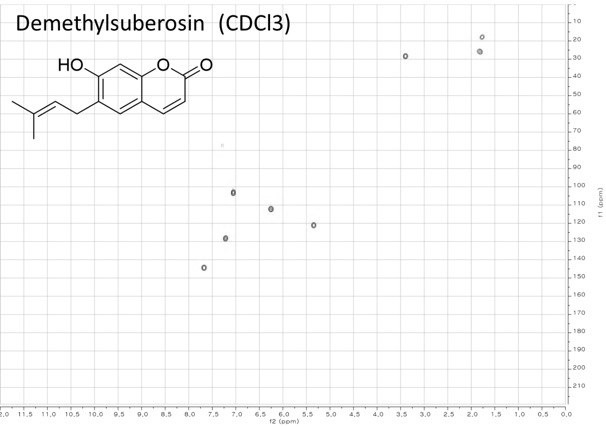


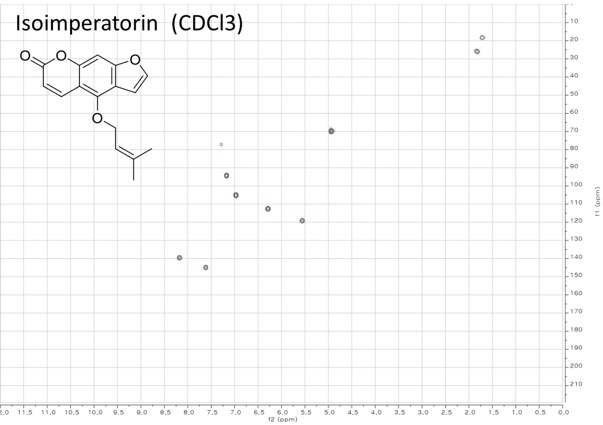


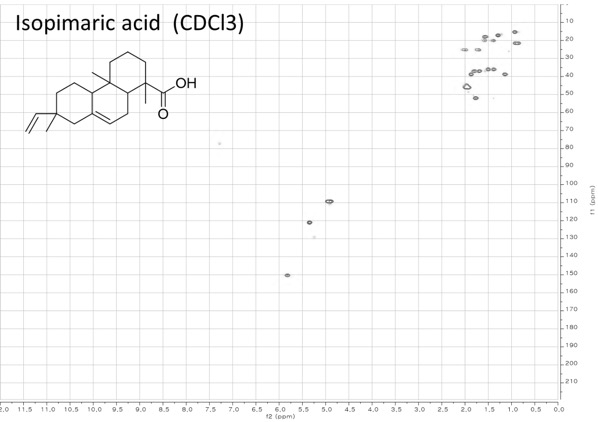


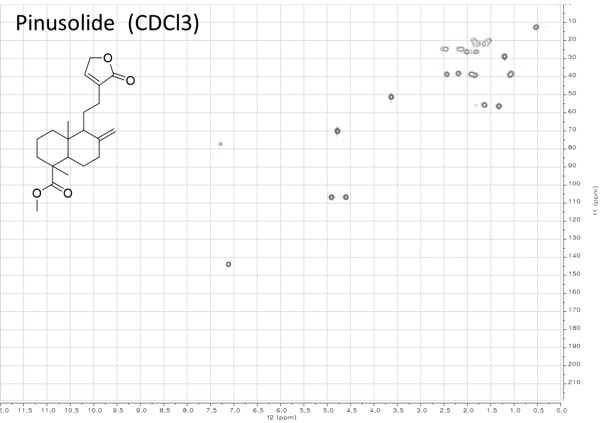


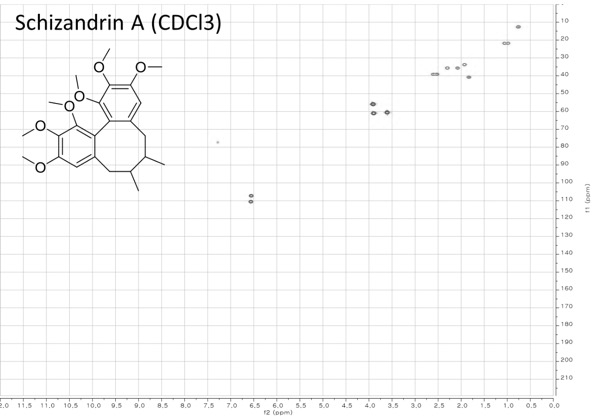


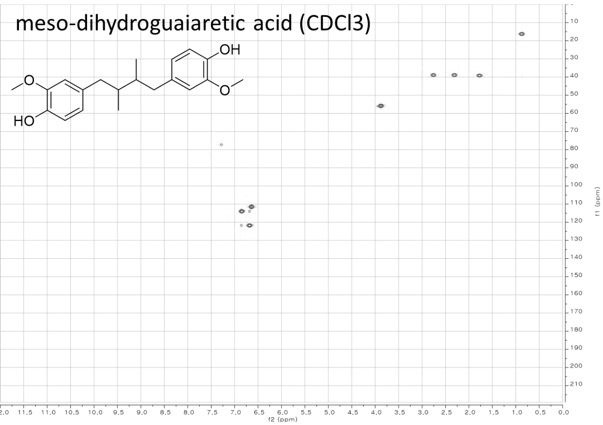


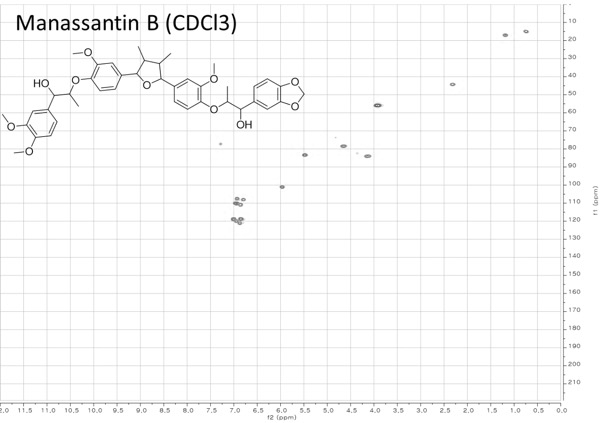


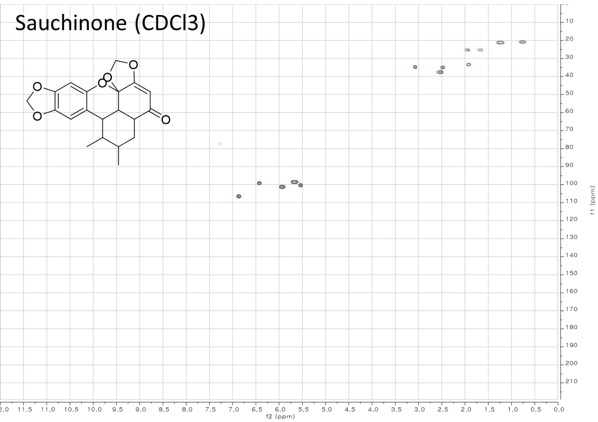


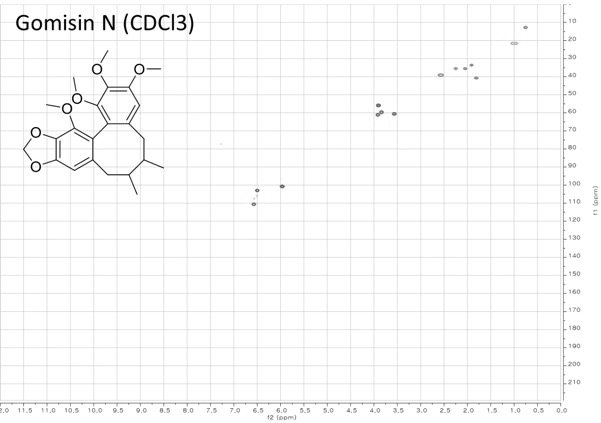


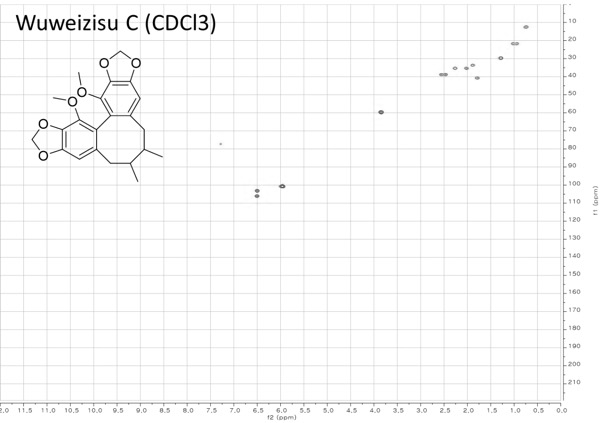


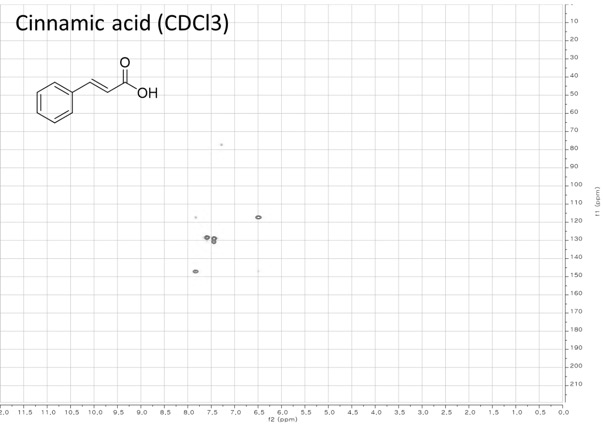


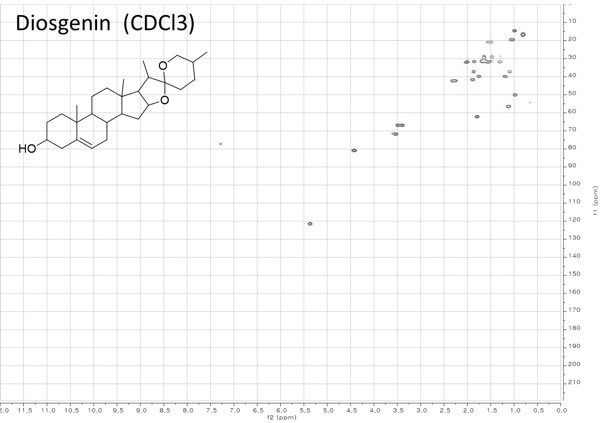


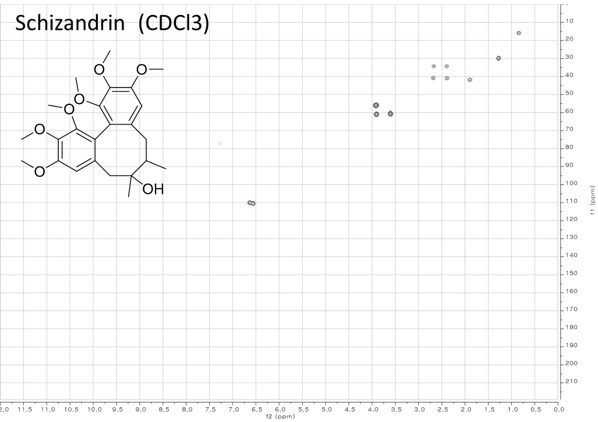


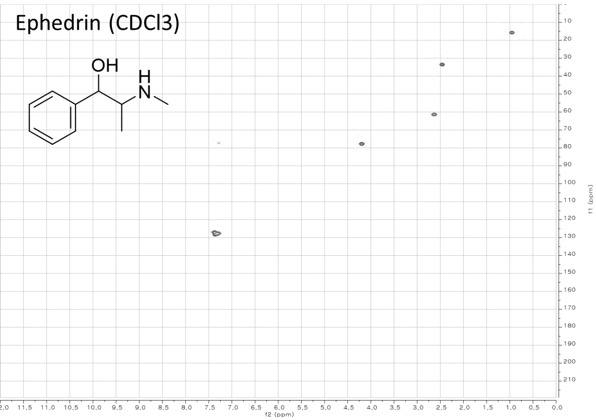


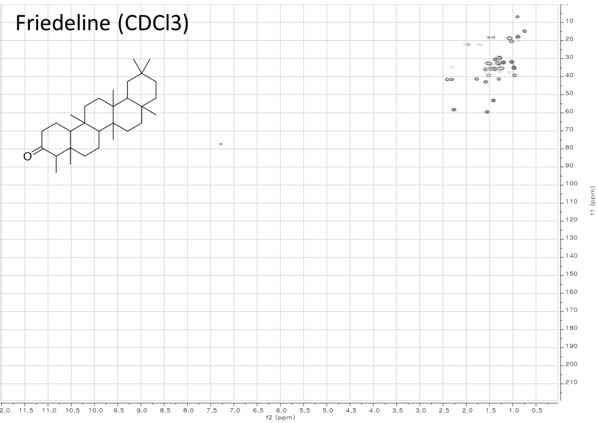


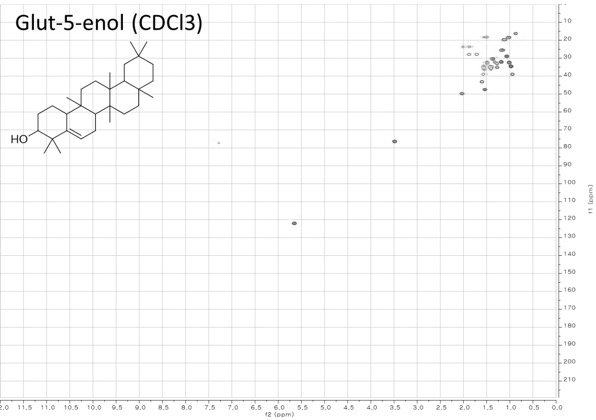


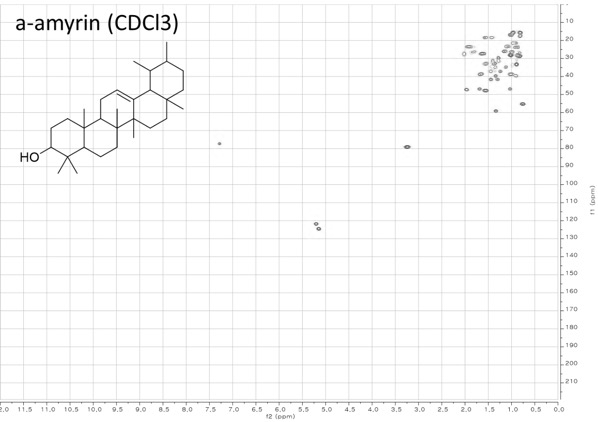


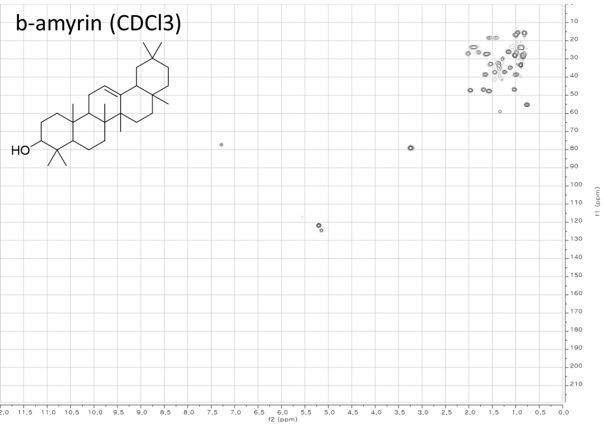


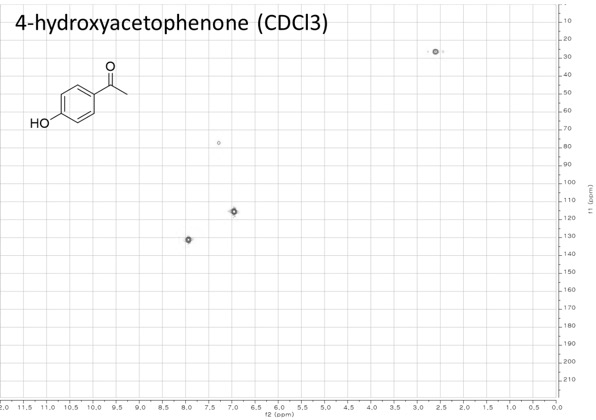


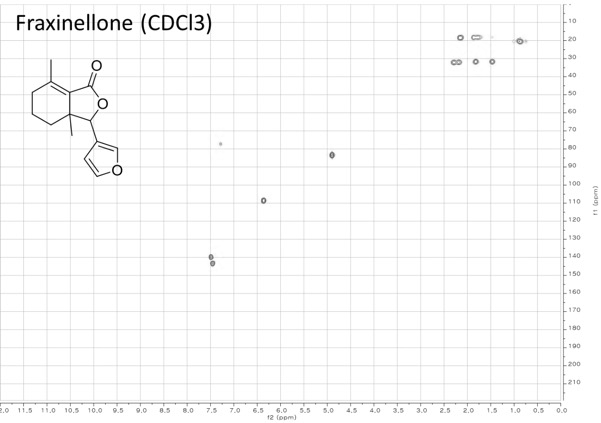


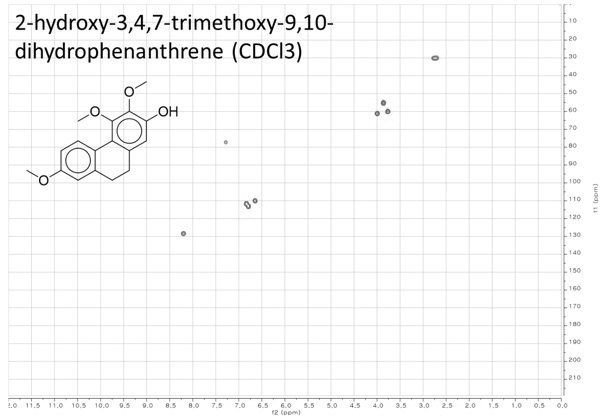


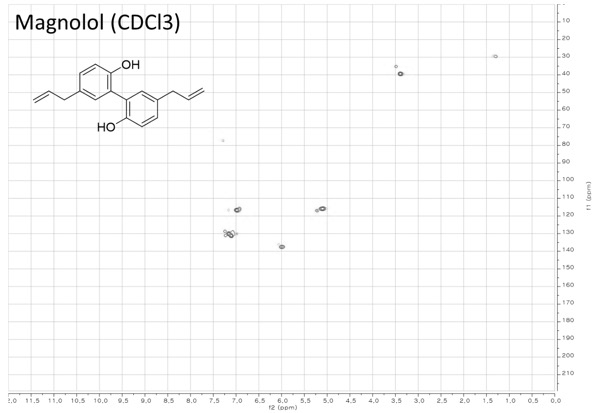


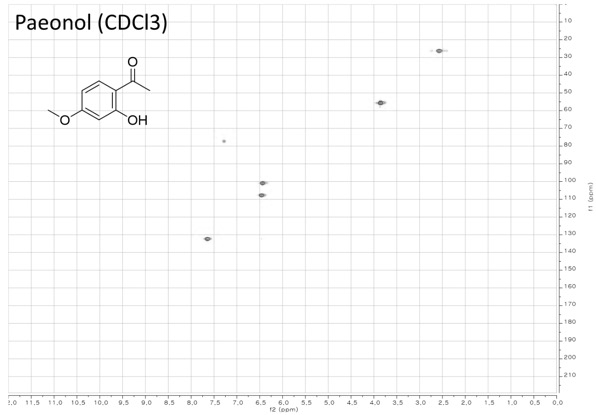


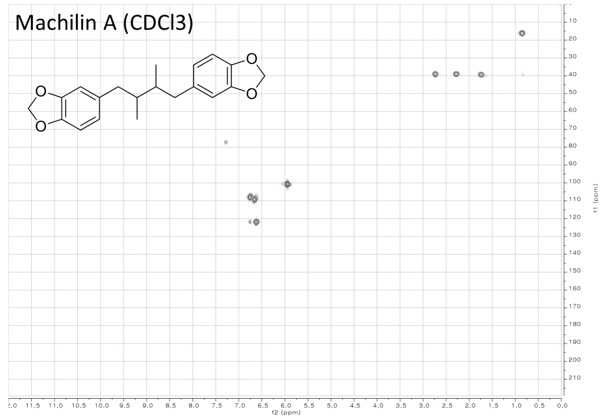


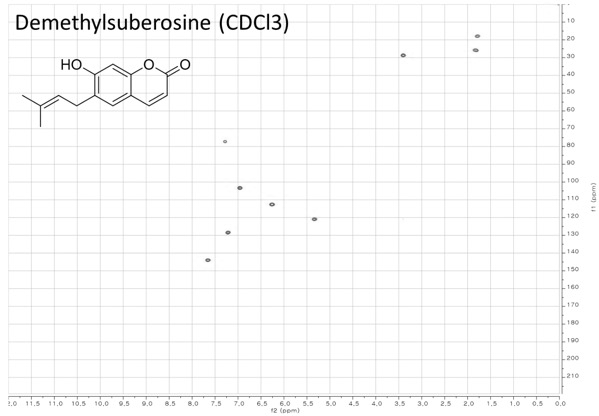


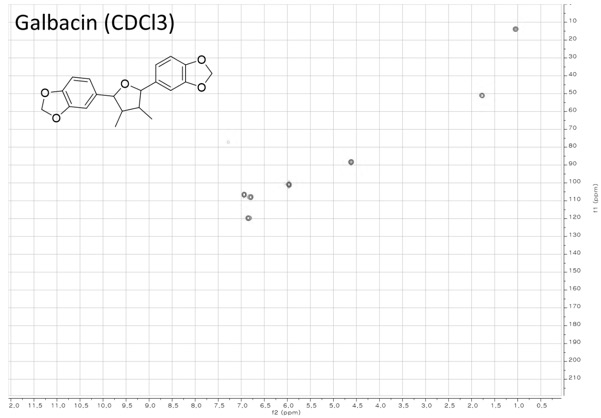


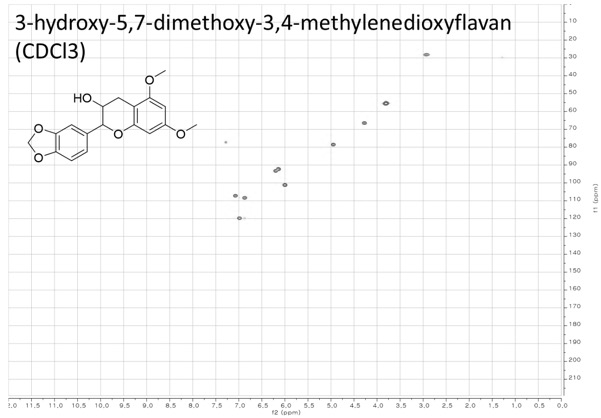


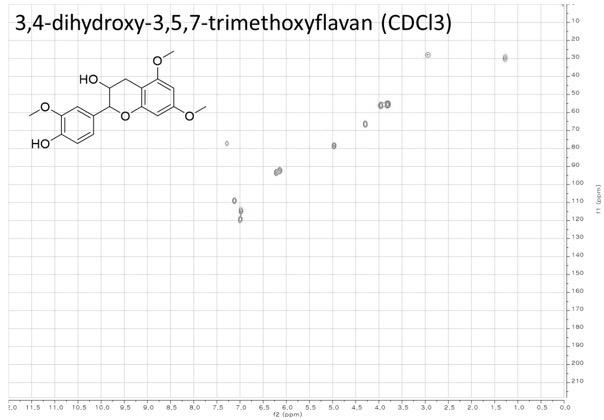


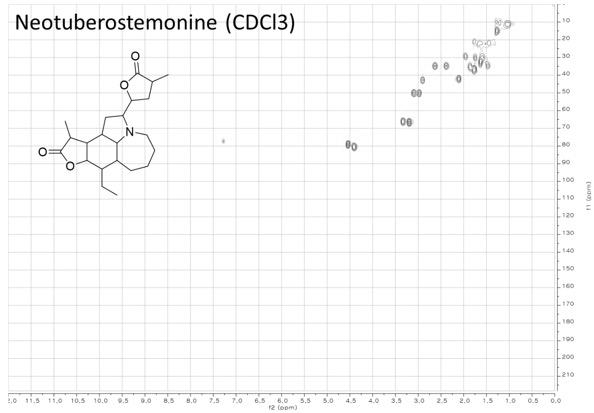


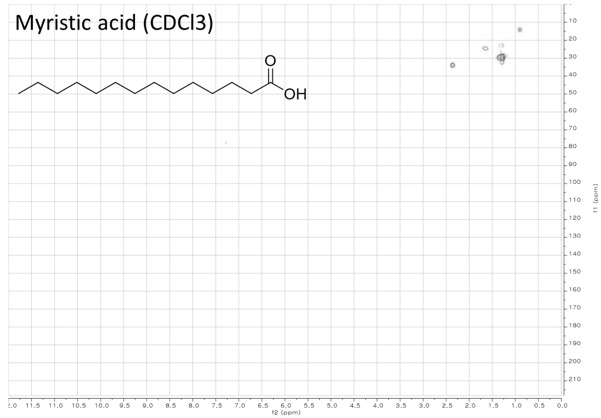


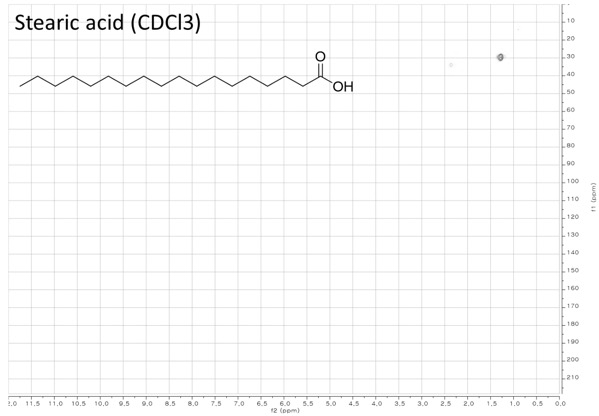


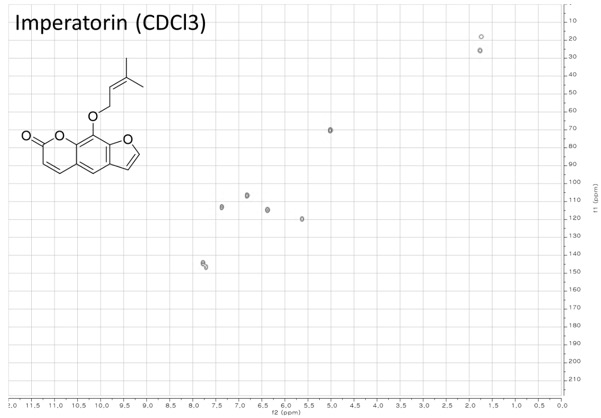


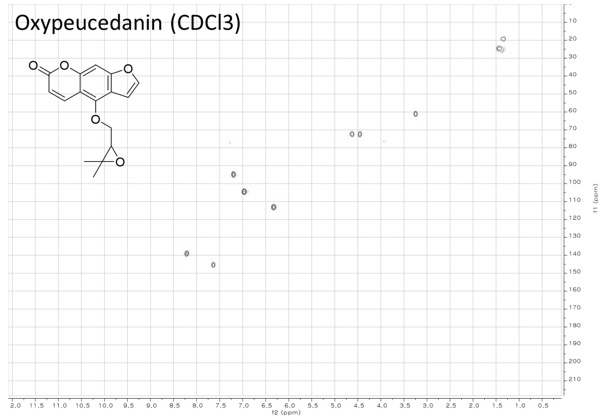


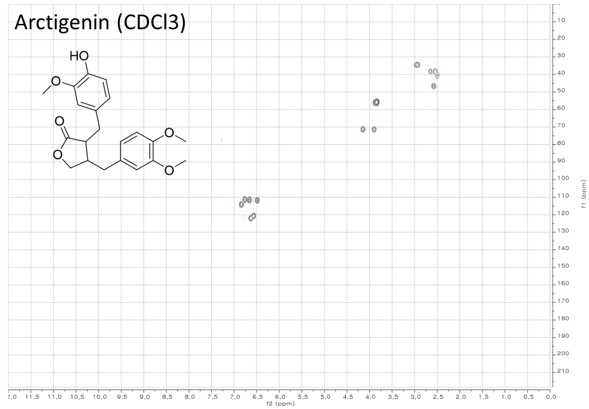


9.2. Experimental HSQC spectra in MeOD


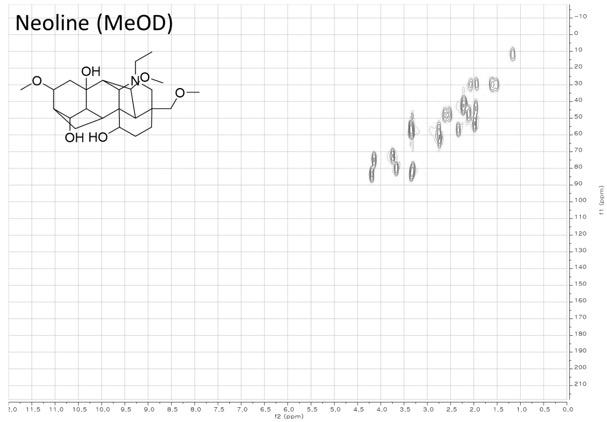


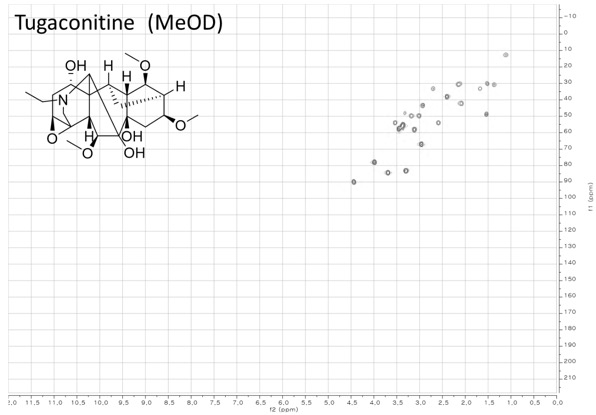


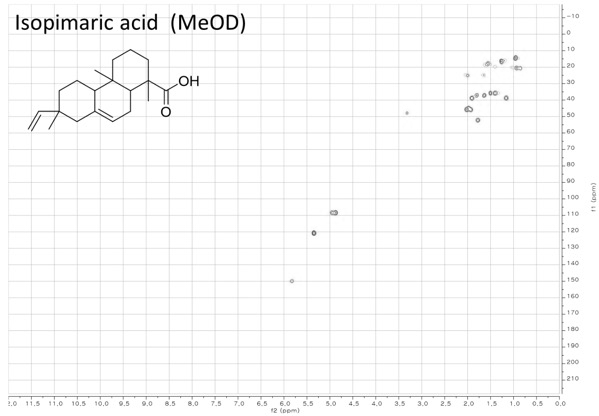


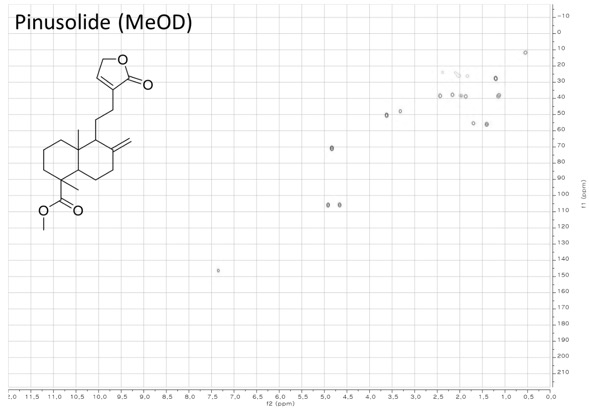


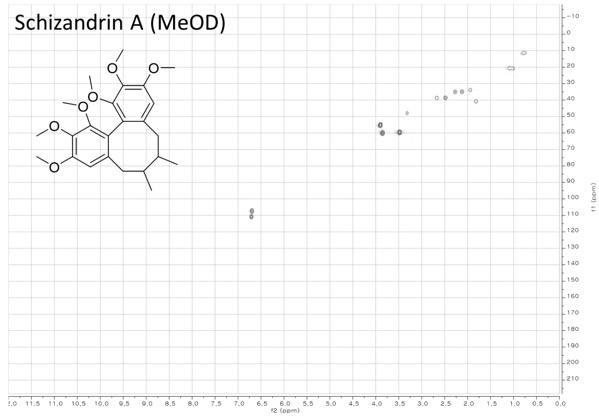


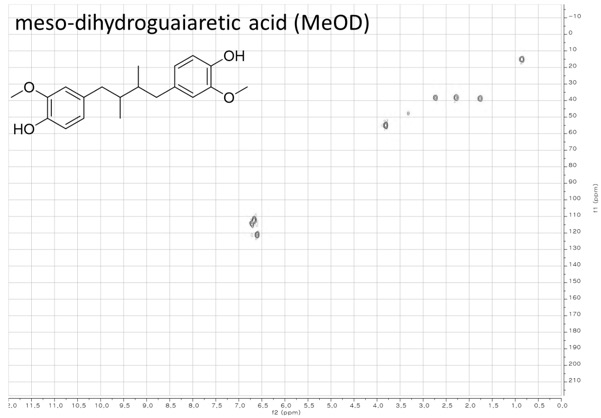


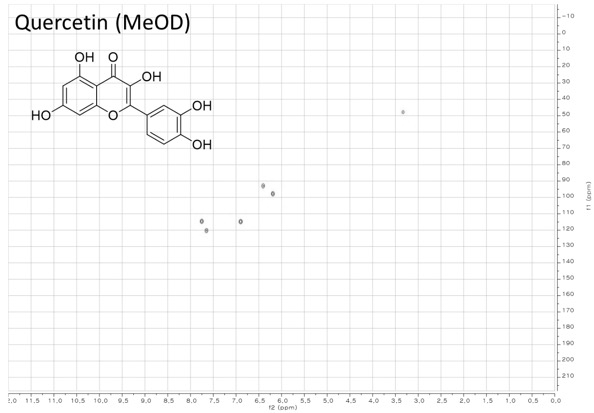


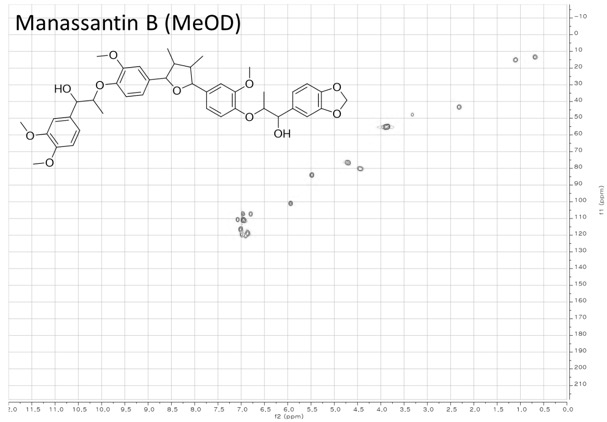


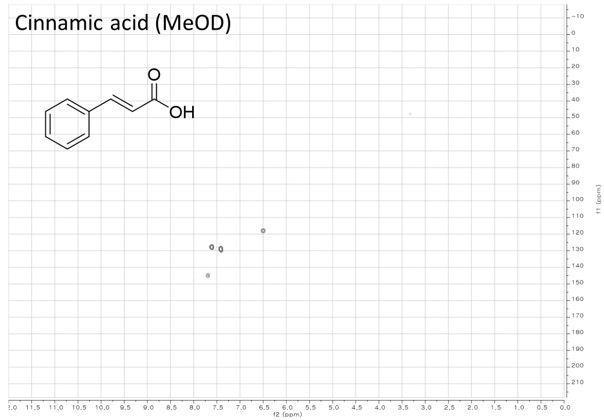


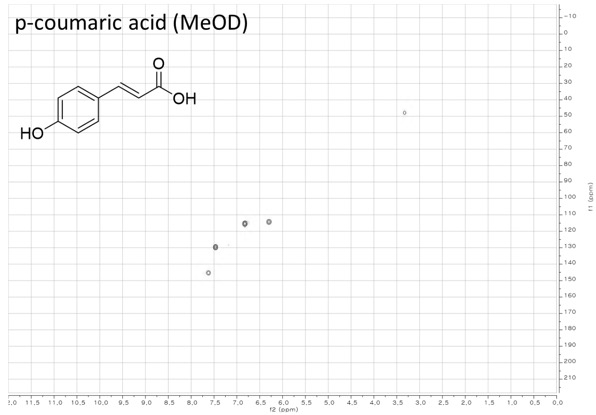


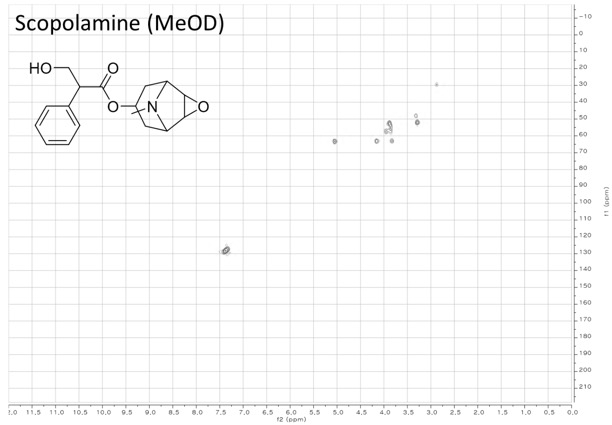


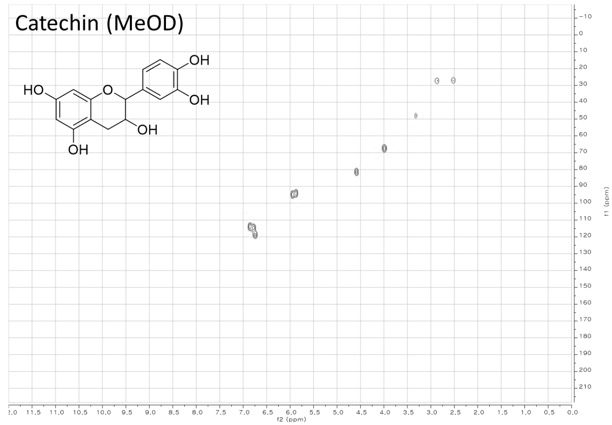


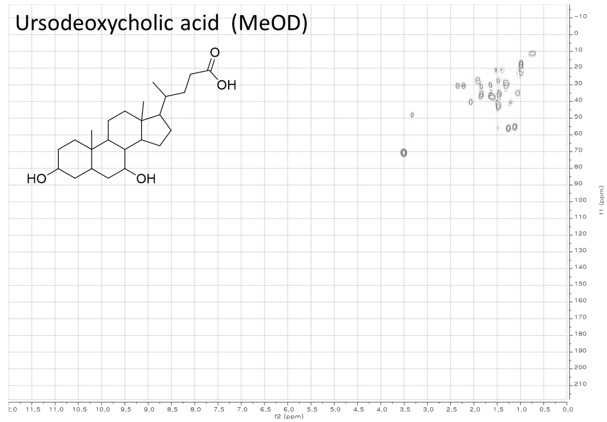


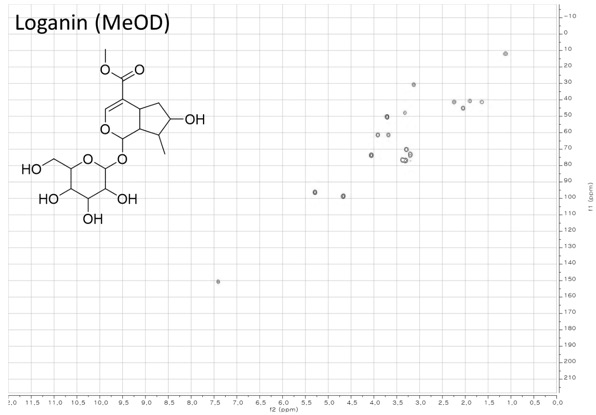


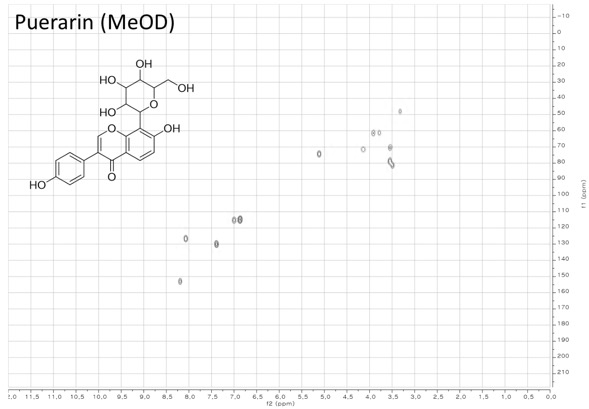


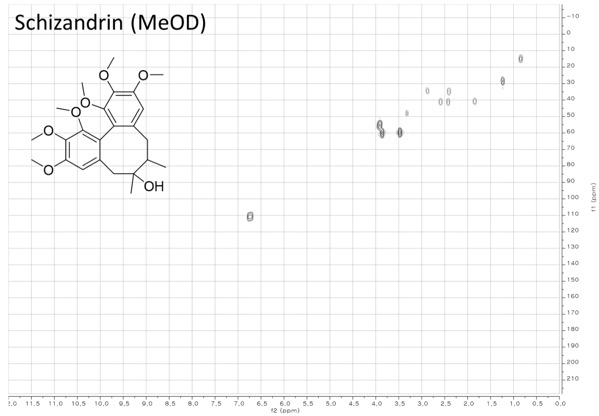


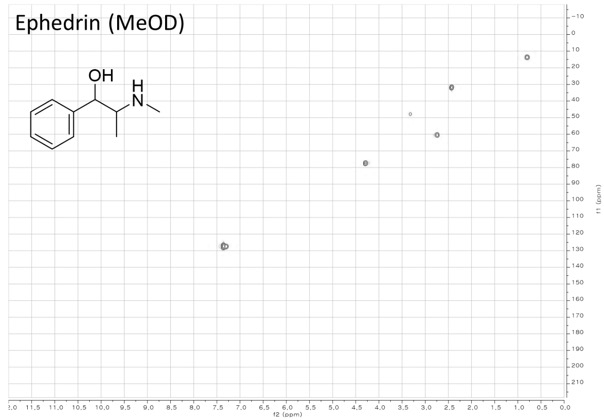


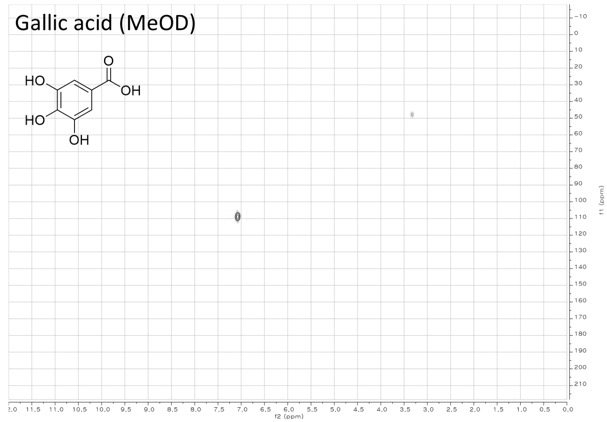


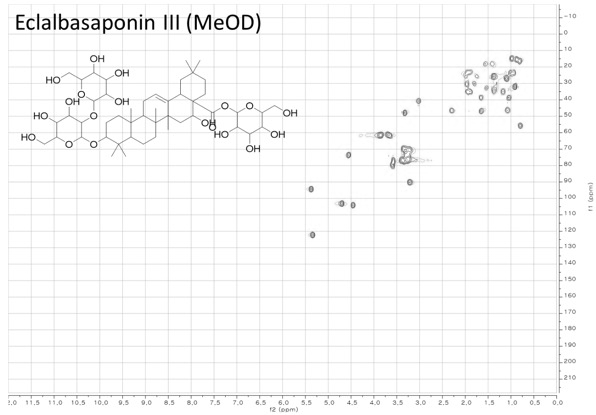


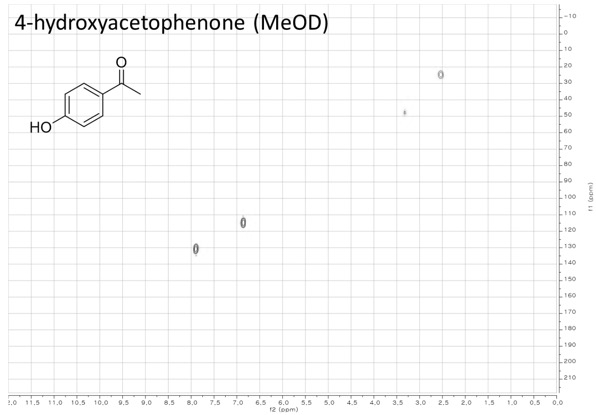


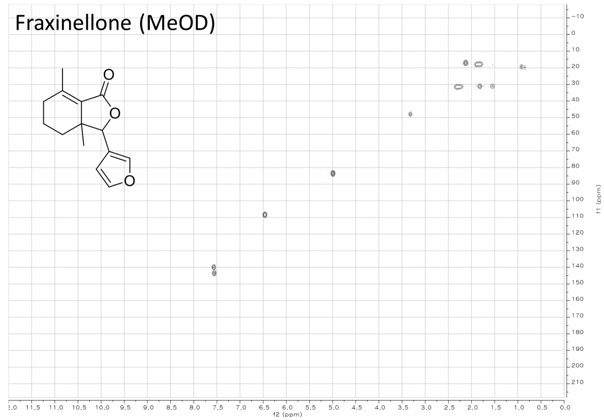


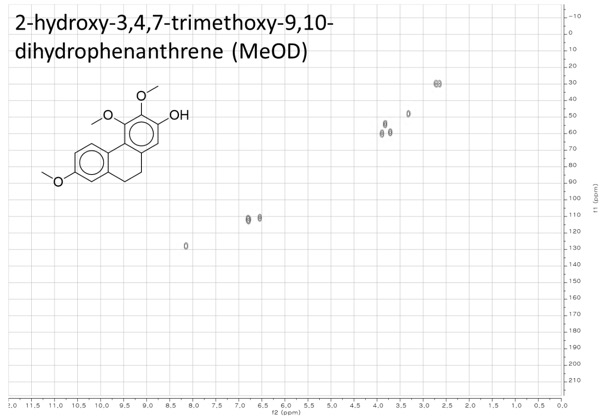


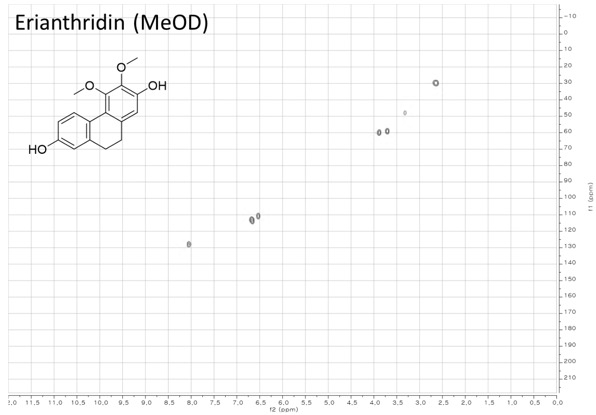


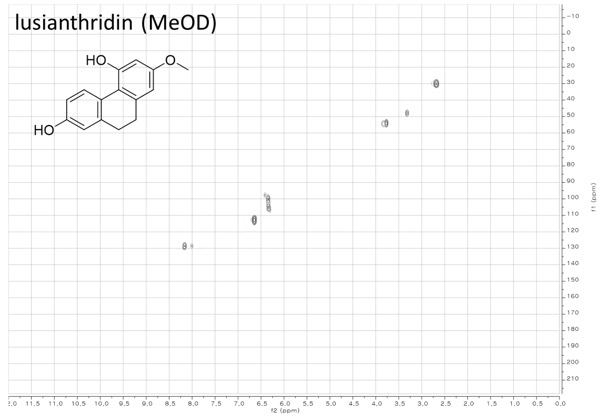


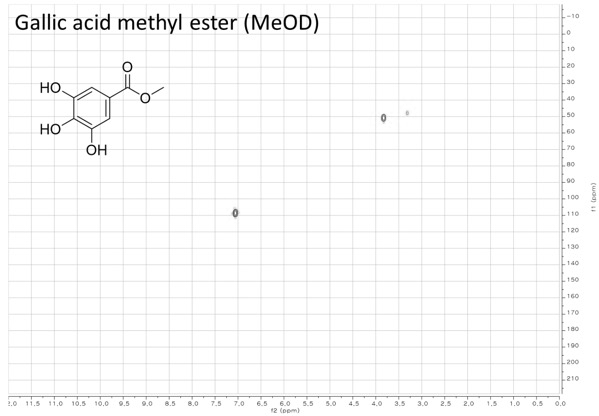


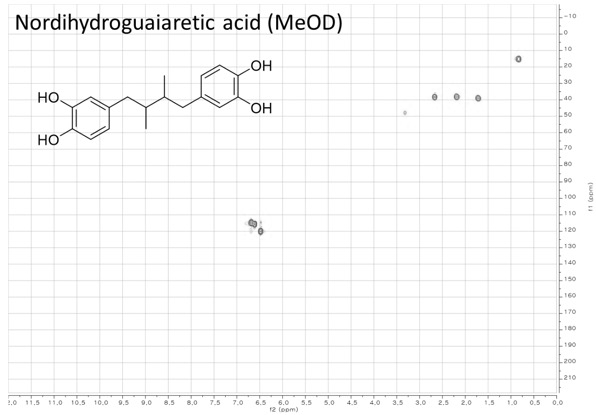


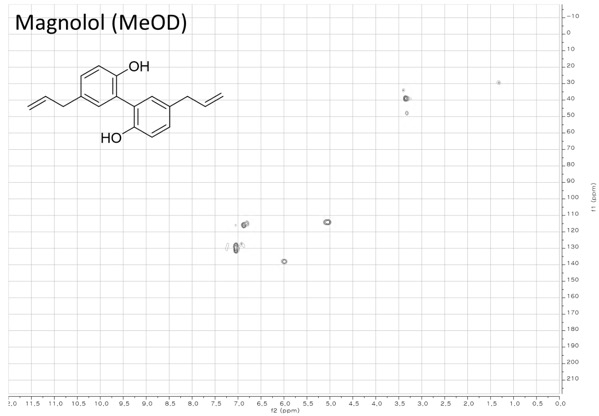


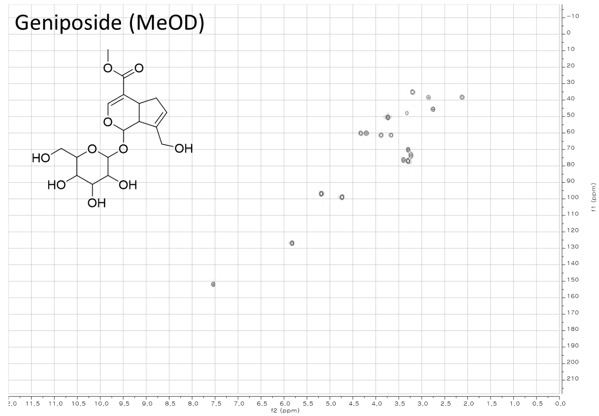


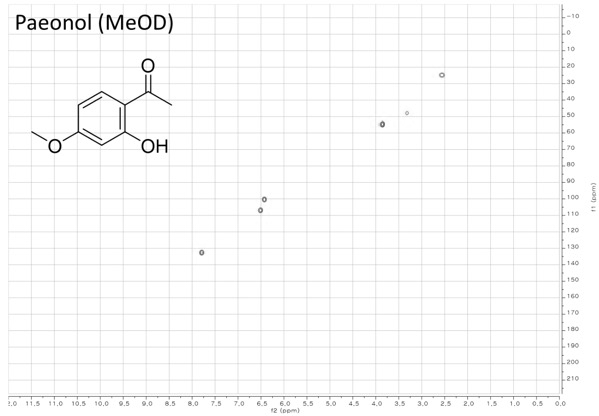


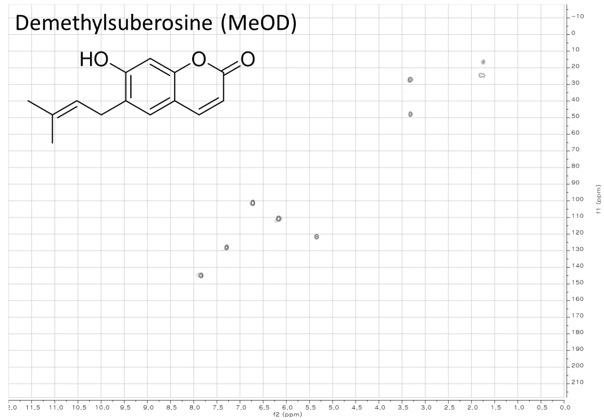

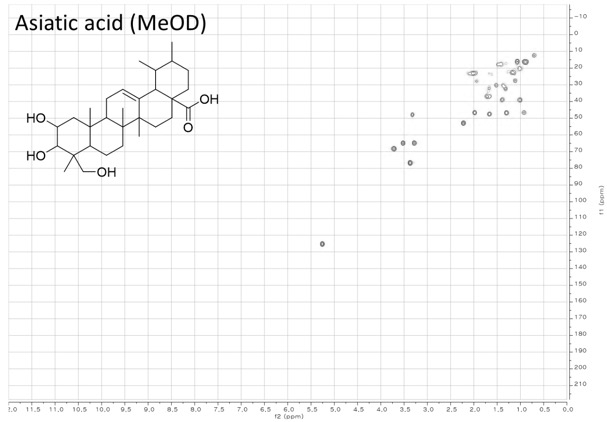


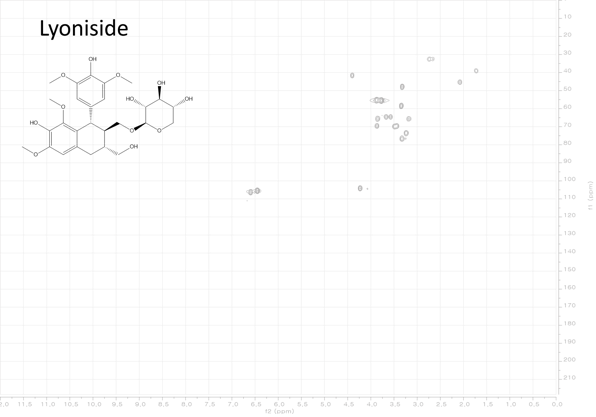


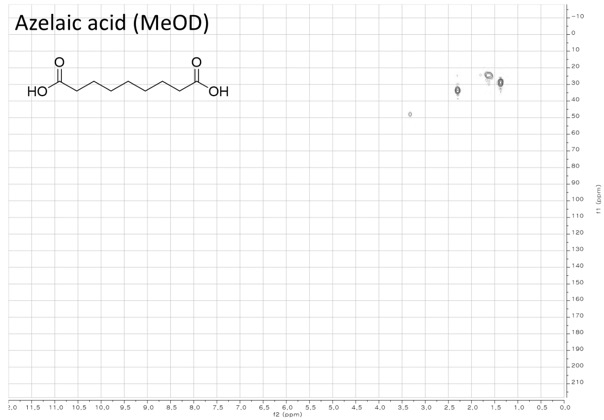


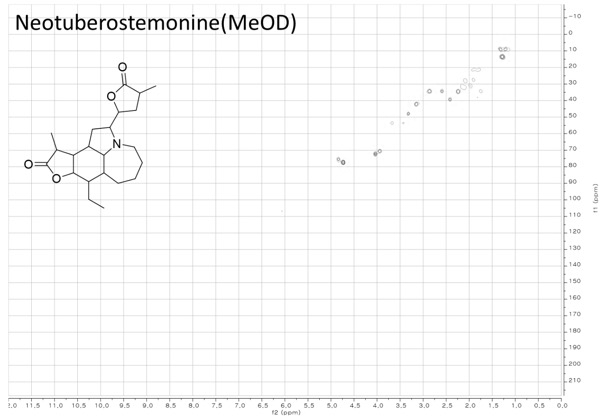


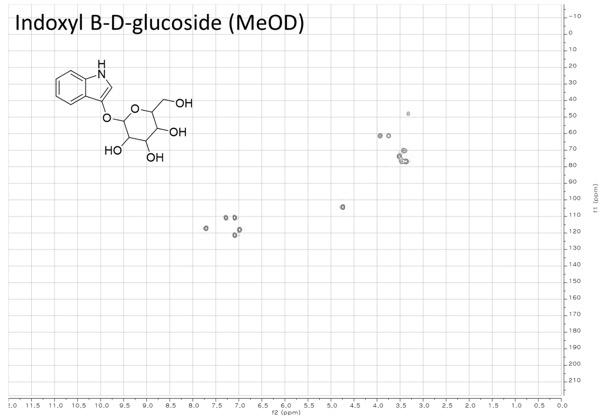


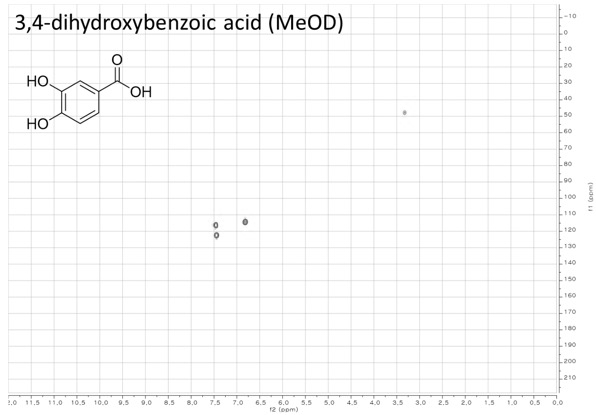


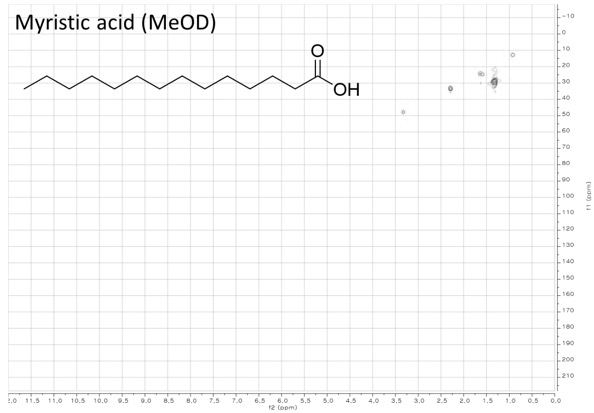


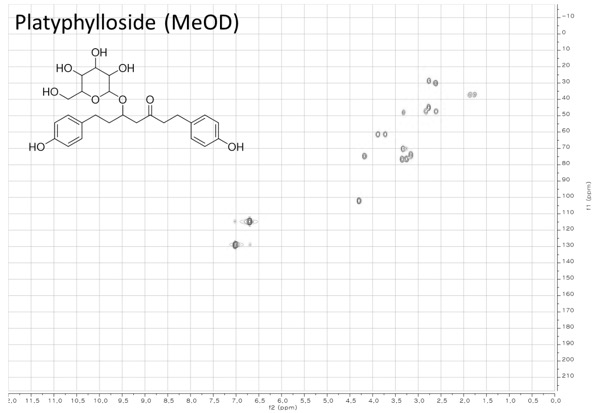


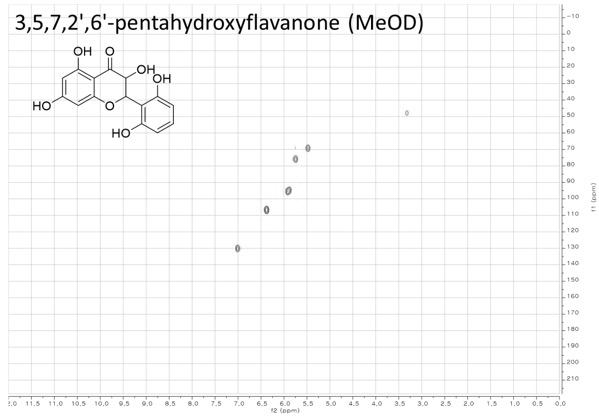


**10. Structure elucidation of previously undescribed compounds from *A. pilosa* and *Dictyota sp.***

**10.1. ^1^H and ^13^C NMR data of compounds 1 and 2**

| 1,2,3-trihydroxyursa-12,18-dien-28-oic acid (**1**) | | | 14,15-dihydroxy acutilol A 8-acetate (**2**) | | |
| --- | --- | --- | --- | --- | --- |
| position | δC | δH (J in Hz) | position | δC | δH (J in Hz) |
| 1 | 81.5 | 3.63 (1H, brd, *J* = 4.6 Hz) | 1 | 139.8 |  |
| 2 | 76.5 | 4.51 (1H, dd, *J* = 4.6, 2.8 Hz) | 2 | 36.7 | 2.88 (2H, brs) |
| 3 | 77.7 | 3.51 (1H, d, *J* = 2.8 Hz) | 3 | 124.4 | 5.38 (1H, brs) |
| 4 | 39.4 |  | 4 | 144.1 |  |
| 5 | 54.2 | 0.96 (1H, d, *J* = 11.2 Hz) | 5 | 60.9 | 3.33 (1H, m) |
| 6 | 18.5 | 1.70 (1H, m) 1.62 (1H, m) | 6 | 72.1 | 3.94 (1H, dd, *J* = 9.3 Hz) |
| 7 | 35.7 | 1.67 (1H, m) 1.58 (1H, m) | 7 | 48.9 | 1.41 (1H, m) |
| 8 | 40.3 |  | 8 | 71.5 | 5.22 (1H, td, *J* = 8.0, 3.4 Hz) |
| 9 | 49.8 | 1.90 (1H, m) | 9 | 40.1 | 2.48 (2H, d, *J* = 8.0 Hz) |
| 10 | 44.2 |  | 10 | 125.4 |  |
| 11 | 27.3 | 3.44 (1H, m) 2.45 (1H, m) | 11 | 31.5 | 1.86 (1H, m) |
| 12 | 127.9 | 5.88 (1H, dd, *J* = 5.6, 2.4 Hz) | 12 | 32.6 | 1.56 (1H, m) 1.37 (1H, m) |
| 13 | 139 |  | 13 | 28.9 | 1.52 (1H, m) 1.29 (1H, m) |
| 14 | 45.5 |  | 14 | 79.7 | 3.25 (1H, brd, *J* = 9.3 Hz) |
| 15 | 29.4 | 2.37 (1H, m) 1.35 (1H, m) | 15 | 74 |  |
| 16 | 36.1 | 2.63 (1H, d, *J* = 13.2 Hz) 1.58 (1H, m) | 16 | 25.1 | 1.13 (3H, s) |
| 17 | 50.2 |  | 17 | 20.7 | 1.70 (3H, s) |
| 18 | 135.2 |  | 18 | 16 | 1.87 (3H, s) |
| 19 | 135.2 |  | 19 | 17.7 | 0.89 (3H, d, *J* = 6.6 Hz) |
| 20 | 35.2 | 2.25 (1H, m) | 20 | 25.7 | 1.15 (3H, s) |
| 21 | 27.4 | 2.25 (1H, m) 1.39 (1H, m) | 21 | 173 |  |
| 22 | 32.3 | 2.20 (1H, m) 1.78 (1H, m) | 22 | 21.5 | 2.08 (3H, s) |
| 23 | 18.4 | 1.40 (3H, s) |  |  |  |
| 24 | 30.8 | 1.29 (3H, s) |  |  |  |
| 25 | 13.5 | 1.63 (3H, s) |  |  |  |
| 26 | 19.2 | 1.20 (3H, s) |  |  |  |
| 27 | 22.6 | 1.23 (3H, s) |  |  |  |
| 28 | 179.1 |  |  |  |  |
| 29 | 20.1 | 1.90 (3H, s) |  |  |  |
| 30 | 19.3 | 1.13 (3H, d, *J* = 6.9 Hz) |  |  |  |

**10.2. 1,2,3-trihydroxyursa-12,18-dien-28-oic acid (1) from *A. pilosa***

**10.2.1. Structure elucidation of 1,2,3-trihydroxyursa-12,18-dien-28-oic acid (1)**

Compound **1** was isolated with white amorphous powder. The molecular formula of C_30_H_46_O_5_ was determined by high-resolution electospray ionization mass spectrometry (HRESIMS) ion peak at *m*/*z* 485.6750 [M – H]^-^ (calcd for C­_30_H_45_O_5_, 485.6753). Two of eight degrees of unsaturation from molecuar formula were assigned to carbon-carbon double bonds (*δ*_C_ 139.0 (C-13), 135.2 (C-19), 135.2 (C-18), and 127.9 (C-12)), one was from an carboxyl carbonyl (*δ*_C_ 170.1). Thus, the remaining five degrees of unsaturation were accommodated from a pentacyclic carbon skeleton. ^1^H NMR spectrum of **1** showed six single methyl group signals at *δ*_H_ 1.90 (3H, s, H-29), 1.63 (3H, s, H-25), 1.40 (3H, s, H-23), 1.29 (3H, s, H-24), 1.23 (3H, s, H-27), and 1.20 (3H, s, H-26) and one doublet methyl group signal at *δ*_H_ 1.13 (3H, d, *J* = 6.9 Hz, H-30) along with three proton signals at *δ*_H ­_4.51 (1H, dd, *J* = 4.6, 2.8 Hz, H-2), 3.63 (1H, d, *J* = 4.5 Hz, H-1), and 3.51 (1H, d, *J* = 2.8 Hz, H-3) which was similar to those of goreishic acid I with one more hydroxyl group. By further analysis of ^1^H-1^1^H COSY and ^1^H-^13^C HMBC correlation, compound 1 was assigned as 1,2,3-trihydroxyursa-12,18-dien-28-oic acid.

Key ^1^H-^1^H COSY and HMBC correlations of compound 1

**10.2.2. NMR spectra of compound 1**

^1^H NMR spectrum of compound 1 (600 MHz, Pyridine-*d*4)

^13^C NMR spectrum of compound 1 (150 MHz, pyridine-*d*4)

^1^H-^13^C HSQC spectrum of compound 1 (600 MHz, pyridine-*d­*4)

^1^H-^1^H COSY spectrum of compound 1 (600 MHz, pyridine-*d­*4)

^1^H-^13^C HMBC spectrum of compound 1 (600 MHz, pyridine-*d*4)

**10.3. 14,15-dihydroxy acutilol A 8-acetate (2) from *Dictyota sp.***

**10.3.1. Structure elucidation of 14,15-dihydroxy acutilol A 8-acetate (2)**

Compound **2** was obtained as pale brownish amorphous solid and its molecular formula was C_22_H_36_O_5_ which was derived from HRESIMS ion peak at *m*/*z* 381.5262 [M + H]^+^ (calcd for C_22_H_37_O_5_, 381.5262). Two of five degrees of unsaturation from molecular formula were assigned to carbon-carbon double bonds (*δ*_C_ 144.1 (C-4), 139.8 (C-1), 125.4 (C-10), and 124.4 (C-3)) and one was from an ester carbonyl (*δ*_C_ 173.0). Thus, the remaining two degrees of unsaturation were derived from a bicyclic carbon skeleton. The ^1^H-^13^C HMBC correlation between *δ*_H_ 2.08 (3H, s) and *δ*_C_ 173.0 suggested the presence of an acetate ester. By analysis of ^1^H-1^1^H COSY and ^1^H-^13^C HMBC correlation, the molecular structure of compound **2** was similar to that of acutilol A acetate, a pachydictyane diterpenoid, but the acetate ester was substituted to C-8 and molecular formula indicated that compound 2 had two more oxygen atoms. From those results, compound **2** was assigned to 14,15-dihydroxy acutilol A 8-acetate.

Key ^1^H-^1^H COSY and HMBC correlations of compound 2

**10.3.2. NMR spectra of compound 2**

^1^H NMR spectrum of compound 2 (600 MHz, methanol-*d­*4)

^13^C NMR spectrum of compound 2 (600 MHz, methanol-*d*4)

^1^H-^13^C HSQC spectrum of compound 2 (600 MHz, methanol-*d*4)

^1^H-^1^H COSY spectrum of compound 2 (600 MHz, methanol-*d*4)

^1^H-^13^C HMBC spectrum of compound 2 (600 MHz, methanol-*d*4)
